# Molecular basis underlying the successful invasion of hexaploid cytotypes of *Solidago canadensis *L.: Insights from integrated gene and miRNA expression profiling

**DOI:** 10.1002/ece3.5084

**Published:** 2019-03-26

**Authors:** Chanchan Xu, Yimeng Ge, Jianbo Wang

**Affiliations:** ^1^ State Key Laboratory of Hybrid Rice, College of Life Sciences Wuhan University Wuhan China

**Keywords:** invasiveness, miRNA, ploidy, polyploid, *Solidago canadensis* L., transcriptome

## Abstract

Dissecting complex connections between cytogenetic traits (ploidy levels) and plant invasiveness has emerged as a popular research subject in the field of invasion biology. Although recent work suggests that polyploids are more likely to be invasive than their corresponding diploids, the molecular basis underlying the successful invasion of polyploids remains largely unexplored. To this end, we adopted an RNA‐seq and sRNA‐seq approach to describe how polyploids mediate invasiveness differences in two contrasting cytotypes of *Solidago canadensis* L., a widespread wild hexaploid invader with localized cultivated diploid populations. Our analysis of the leaf transcriptome revealed 116,801 unigenes, of which 12,897 unigenes displayed significant differences in expression levels. A substantial number of these differentially expressed unigenes (DEUs) were significantly associated with the biosynthesis of secondary metabolites, carbohydrate metabolism, lipid metabolism, and environmental adaptation pathways. Gene Ontology term enrichment‐based categorization of DEU‐functions was consistent with this observation, as terms related to single‐organism, cellular, and metabolic processes including catalytic, binding, transporter, and enzyme regulator activity were over‐represented. Concomitantly, 186 miRNAs belonging to 44 miRNA families were identified in the same leaf tissues, with 59 miRNAs being differentially expressed. Furthermore, we discovered 83 miRNA‐target interacting pairs that were oppositely regulated, and a meticulous study of these targets depicted that several unigenes encoding transcription factors, DNA methyltransferase, and leucine‐rich repeat receptor‐like kinases involved in the stress response were greatly influenced. Collectively, these transcriptional and epigenetic data provide new insights into miRNA‐mediated gene expression regulatory mechanisms that may operate in hexaploid cytotypes to favor successful invasion.

## 1 INTRODUCTION

Amid growing evidence of biological invasion impacts on global biodiversity, ecosystem functioning, species conservation, and even social and economic activities (Dyer et al., 2017; Rejmánek, 2015), there is mounting interest in searching the determining elements underlying the successful invasion of alien species. A key element associated with successful invasion of alien species is their capacity for rapid adaptation to environmental challenges following introduction (Huang et al., 2017). Identifying critical traits that benefit this rapid environmental adaptation is therefore a talking point for conservation concern as it can create greater opportunities to predict the invasion risk related to diverse alien species. A steady stream of ecological research in recent years has identified a variety of shared ecological traits related to invasiveness among invasive alien species, such as increased growth and fecundity, wide ecological tolerance, high fitness, and strong clonal propagation (Richardson & Pyšek, 2008). As ecological traits have not generated an explicit recognition pattern in plant invasiveness, some invasion biologists have switched from ecological traits to genetic or genomic traits in comprehending patterns of plant invasiveness (Hodgins, Lai, Nurkowski, Huang, & Rieseberg, 2013; Prentis & Pavasovic, 2013; Rius & Darling, 2014). For instance, Rius and Darling (2014) showed that genetic admixture may act as a genuine driver to play central roles in the successful invasion of genetically admixed individuals. Likewise, another related study performed on invasive alien plants showed a statistical connection between ploidy level and invasiveness, concluding that polyploid plants were more likely to be invasive than diploids and that ploidy level (and chromosome number) was positively related to plant invasiveness (Pandit, Pocock, & Kunin, 2011; Pandit, White, & Pocock, 2014).

Polyploids, recognized as organisms that possess more than two complete sets of chromosomes in their somatic cells, have often been suggested to represent a powerful driver in the speciation, evolution, and adaptation of plants, with far‐reaching ecological and evolutionary consequences. On the other hand, polyploids often appear to be over‐represented in invasive plants (Thébault, Gillet, Müller‐Schärer, & Buttler, 2011) and have cumulatively been acknowledged as a latent advantageous attribute of plant invaders (Pandit et al., 2011; te Beest et al., 2012). Furthermore, the influences of polyploids can act as a cascade process that directly or indirectly mediates virtually all aspects of plant genetics, morphology, physiology, life history, and ecology (Levin, 1983). Therefore, many evolutionary biologists believe that polyploids provide introduced plants with new features that permit them to invade largely varied environments or expand their geographical range. However, this hypothesis has not yet been proved efficiently. Ecological studies have conventionally focused on comparing clearly relevant diploid and polyploid in their native and introduced ranges (Hahn, Buckley, & Müller‐Schärer, 2012), but few studies have elucidated the molecular basis underlying the invasiveness difference in alien plants with different ploidy levels (cytotypes). Therefore, the genetic and epigenetic impact imposed by ploidy alteration remains elusive.

Studies on natural and synthetic polyploids have repeatedly revealed that rapid and dynamic changes at the genetic, gene expression, and epigenetic levels occur after polyploid formation (Chen, 2007; Jackson & Chen, 2010; Sun, Wu, et al., 2017). Likewise, evidence is also mounting that epigenetic modifications can change gene expression and reconstruct gene expression networks thus resulting in pronounced phenotypic alterations (Hao, Lucero, Sanderson, Zacharias, & Holbrook, 2013; Song & Chen, 2015) and allowing polyploids to occupy new habitats, grow vigorously and improve adaptation in novel environments (Madlung, 2013). As an extensive type of epigenetic modifications in nonmodel organisms, miRNA has attracted considerable concern due to its regulatory mechanisms for gene expression. Additionally, miRNA is highly conserved in evolution but becomes activated in polyploidization (Axtell, 2008; Ha et al., 2009). More importantly, alterations in miRNA expression can mediate their target‐gene expression at the post‐transcriptional level, and this effect is viewed as one of the main reasons for phenotypic changes of polyploids (Chen, 2007; Ha et al., 2009). Accordingly, elucidating the divergences in gene and miRNA expression between different ploidy levels (cytotypes) of alien plants and how they influence phenotypic differentiation is crucial to explain how polyploids might have contributed to successful invasion.

Herein, we evaluate the effect of polyploids on gene and miRNA expression while also considering the potential roles of miRNA‐mediated gene expression regulation in driving differences in invasiveness between diploid and hexaploid cytotypes of *Solidago canadensis *L. Specifically, the objectives of our current work are as follows: (a) to characterize the initial expression profiling of genes and miRNAs in two identified ploidy levels, that is, diploid and hexaploid cytotypes of *S. canadensis* at a genome‐wide scale; (b) to determine key candidate genes and miRNA regulators that may contribute to the successful invasion of hexaploid cytotypes based on gene and miRNA expression divergences, as well as over‐represented functional categories of these candidates; and (c) finally to explore the strong evidence for the potential genetic roles of epigenetic and transcriptional alterations in the successful invasion of hexaploid cytotypes. To this end, we adopted an RNA‐seq and sRNA‐seq approach to investigate the divergences of gene and miRNA expression between diploid and hexaploid cytotypes of *S. canadensis*. Furthermore, we constructed a co‐expression network of differentially expressed genes and miRNAs to shed light on the regulatory action of miRNAs. Taken together, our work provides new insights into miRNA‐mediated gene expression regulatory mechanisms that may be useful to explain the successful invasion of hexaploid cytotypes.

## 2 MATERIALS AND METHODS

### 2.1 Study species

*Solidago canadensis* (Asteraceae), a perennial weed native to North America (Werner, Bradbury, & Gross, 1980) where it exists in a diploid, tetraploid, or hexaploid cytotype (Melville & Morton, 1982), has invaded a wide geographical range globally, including New Zealand, Australia, Europe, and Asia (Abhilasha, Quintana, Vivanco, & Joshi, 2008; Szymura, Szymura, Wolski, & Swierszcz, 2018). Introduced into eastern China in the 1930s as an ornamental plant, *S. canadensis* began to escape cultivation and spread in the 1980s. Currently, it has become highly abundant and has noticeably affected the diversity and richness of native plant species (Wang, Jiang, Zhou, & Wu, 2018). However, it is worthwhile noting that, only hexaploid cytotypes of *S. canadensis* have long been convincingly reported to occur widely in the introduced range in China and become invasive thus far (Wang, 2016). Their corresponding diploid cytotypes (also called “Huang Ying” in China) were cultivated mainly in Yunnan Province in southwestern China as an important cut‐flower plant. An earlier experiment carried out with common gardens showed that the growth of hexaploid cytotypes of *S. canadensis* was more vigorous than their related diploids, offering clear advantages for the successful invasion of hexaploid cytotypes (Li, 2011). Additionally, the roots, stems, and leaves of hexaploid cytotypes were morphologically and anatomically distinct from their diploids (Wang, 2007). Overall, the contrasting invasive propensities and geographical and phenotypic differentiation between hexaploid cytotypes and their related diploids make *S. canadensis* an excellent study system to answer such questions as how polyploids both affect gene and miRNA expression and alter molecular pathways that may be responsible for the successful invasion of hexaploid cytotypes and whether miRNA plays key roles in reprogramming the transcriptional expression.

### 2.2 Population sampling and chromosome counting

**Figure 1:**
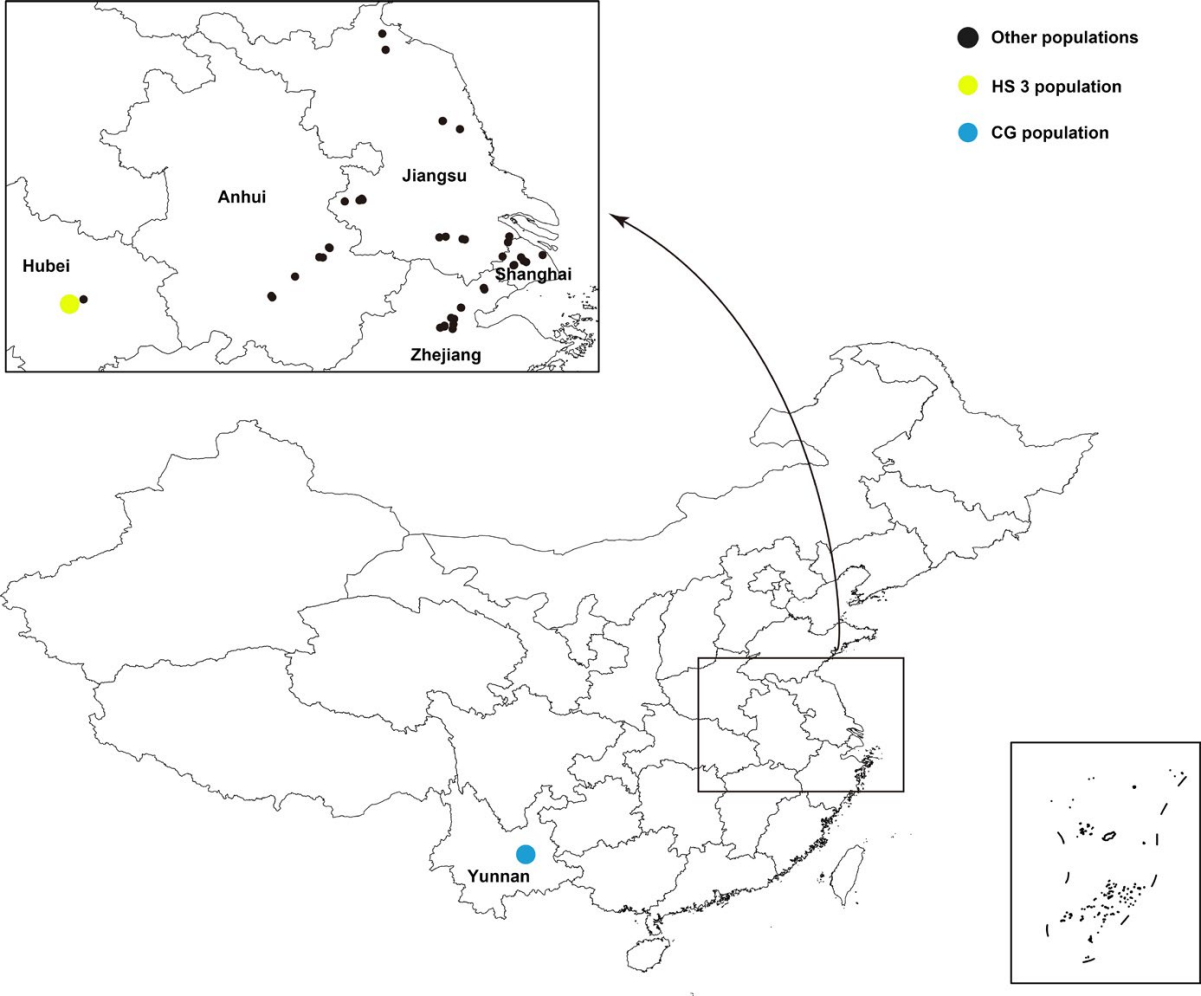
Map of China sampling sites for 43 populations of *S. canadensis* described in Table 1. The circles indicate sampling locations. The blue and yellow circles show the diploid and hexaploid populations used in gene and miRNA expression analyses, respectively

**Figure A1:**
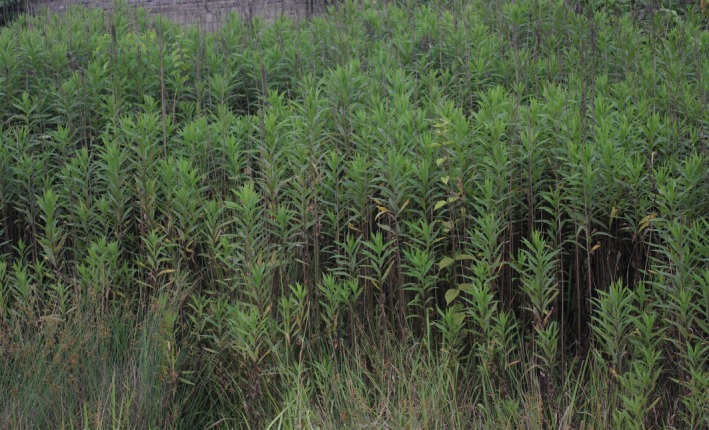
A photograph of one invasive population of *S. canadensis* in eastern China

Invasive populations of *S. canadensis* largely cluster around the Yangtze River Delta, which occupies its main distribution range in China (Figure 1, Figure A1 in Appendix), and cultivated populations were narrowly cultivated in Yunnan Province in southwestern China. Therefore, we sampled invasive populations (separated from each other by at least 3 km) in 42 locations throughout Jiangsu, Zhejiang, Anhui, Hubei, and Shanghai and one cultivated population from Yunnan Province (Table 1; Figure 1). Finally, 449 sampled individuals representing 43 populations were collected and subsequently transplanted into pots with commercial soil and grown for 3 months in the greenhouse of Wuhan University under natural photoperiod conditions. Chromosome counting was performed according to the modified carbol fuchsin squash method (The detailed methods are presented in Supporting Information Appendix S1).

**Table 1:** Geographical coordinates and sample size of 43 populations of *S. canadensis *in China

No.	Population code	Location	Geographical coordinates		Status	No.of samples	Chromosome number
			Latitude (N)	Longitude (E)			
1	MH 1	Minhang District, Shanghai City	N31°08′41.40″	E121°23′19.51″	Invasive	10	54
2	MH 2	Minhang District, Shanghai City	N31°09′54.98″	E121°20′53.35″	Invasive	10	54
3	MH 3	Minhang District, Shanghai City	N31°13′02.09″	E121°18′33.51″	Invasive	10	54
4	SJ 1	Songjiang District, Shanghai City	N31°05′37.02″	E121°11′39.99″	Invasive	10	54
5	SJ 2	Songjiang District, Shanghai City	N31°06′01.76″	E121°12′17.63″	Invasive	10	54
6	PD	Pudong District, Shanghai City	N31°15′14.57″	E121°38′25.04″	Invasive	10	54
7	GY	Guanyun County, Lianyungang City, Jiangsu Province	N34°23′34.49″	E119°14′15.33″	Invasive	8	54
8	XP	Xinpu District, Lianyungang City, Jiangsu Province	N34°38′24.10″	E119°11′06.86″	Invasive	8	54
9	YD 1	Yandu District, Yancheng City, Jiangsu Province	N33°18′14.48″	E120°06′26.04″	Invasive	8	54
10	YD 2	Yandu District, Yancheng City, Jiangsu Province	N33°18′15.30″	E120°06′45.48″	Invasive	8	54
11	DF	Dafeng District, Yancheng City, Jiangsu Province	N33°10′44.02″	E120°22′23.67″	Invasive	8	54
12	TC 1	Taicang City, Jiangsu Province	N31°32′16.26″	E121°07′48.35″	Invasive	10	54
13	TC 2	Taicang City, Jiangsu Province	N31°26′51.43″	E121°06′33.75″	Invasive	10	54
14	KS	Kunshan City, Jiangsu Province	N31°13′49.32″	E121°01′35.66″	Invasive	10	54
15	BH 1	Binhu District, Wuxi City, Jiangsu Province	N31°32′01.06″	E120°09′22.39″	Invasive	10	54
16	BH 2	Binhu District, Wuxi City, Jiangsu Province	N31°29′51.43″	E120°24′50.49″	Invasive	10	54
17	BH 3	Binhu District, Wuxi City, Jiangsu Province	N31°29′27.02″	E120°27′01.11″	Invasive	10	54
18	WJ	Wujin District, Changzhou City, Jiangsu Province	N31°31′26.74″	E120°03′33.15″	Invasive	10	54
19	QX	Qixia District, Nanjing City, Jiangsu Province	N32°06′50.22″	E118°52′17.00″	Invasive	10	54
20	XW 1	Xuanwu District, Nanjing City, Jiangsu Province	N32°05′46.33″	E118°53′07.09″	Invasive	10	54
21	XW 2	Xuanwu District, Nanjing City, Jiangsu Province	N32°05′21.80″	E118°50′13.38″	Invasive	10	54
22	PK	Pukou District, Nanjing City, Jiangsu Province	N32°04′20.65″	E118°36′43.51″	Invasive	10	54
23	JG	Jianggan District, Hangzhou City, Zhejiang Province	N30°17′29.55″	E120°14′22.75″	Invasive	12	54
24	XS 1	Xiaoshan District, Hangzhou City, Zhejiang Province	N30°11′33.37″	E120°16′23.98″	Invasive	10	54
25	XS 2	Xiaoshan District, Hangzhou City, Zhejiang Province	N30°16′30.02″	E120°17′05.09″	Invasive	11	54
26	XS 3	Xiaoshan District, Hangzhou City, Zhejiang Province	N30°07′19.58″	E120°15′41.75″	Invasive	9	54
27	BJ 1	Binjiang District, Hangzhou City, Zhejiang Province	N30°10′18.65″	E120°08′13.93″	Invasive	9	54
28	BJ 2	Binjiang District, Hangzhou City, Zhejiang Province	N30°09′26.83″	E120°08′07.79″	Invasive	12	54
29	XH	Xihu District, Hangzhou City, Zhejiang Province	N30°08′28.35″	E120°04′20.74″	Invasive	10	54
30	NH 1	Nanhu District, Jiaxing City, Zhejiang Province	N30°45′04.50″	E120°44′21.24″	Invasive	13	54
31	NH 2	Nanhu District, Jiaxing City, Zhejiang Province	N30°43′32.96″	E120°44′53.83″	Invasive	10	54
32	HN	Haining City, Zhejiang Province	N30°26′47.84″	E120°23′35.61″	Invasive	9	54
33	JH 1	Jinghu District, Wuhu City, Anhui Province	N31°21′35.87″	E118°22′52.54″	Invasive	12	54
34	JH 2	Jinghu District, Wuhu City, Anhui Province	N31°22′10.33″	E118°22′13.17″	Invasive	12	54
35	SS 1	Sanshan District, Wuhu City, Anhui Province	N31°13′15.10″	E118°13′19.19″	Invasive	12	54
36	SS 2	Sanshan District, Wuhu City, Anhui Province	N31°12′54.60″	E118°16′31.88″	Invasive	12	54
37	LM	Lion Mountain District, Tongling City, Anhui Province	N30°55′23.64″	E117°51′06.76″	Invasive	12	54
38	GC 1	Guichi District, Chizhou City, Anhui Province	N30°36′19.83″	E117°30′10.12″	Invasive	12	54
39	GC 2	Guichi District, Chizhou City, Anhui Province	N30°37′35.69″	E117°29′16.50″	Invasive	12	54
40	HS 1	Hongshan District, Wuhan City, Hubei Province	N30°32′53.42″	E114°31′17.84″	Invasive	7	54
41	HS 2	Hongshan District, Wuhan City, Hubei Province	N30°32′36.65″	E114°24′53.26″	Invasive	9	54
42	HS 3^a^	Hongshan District, Wuhan City, Hubei Province	N30°32′22.40″	E114°25′01.12″	Invasive	14	54
43	CG^a^	Chenggong District, Kunming City, Yunnan Province	N24°55′05.42″	E102°47′51.01″	Cultivated	20	18

a Population used in the analyses of gene and miRNA expression.

### 2.3 Sample preparation, cDNA and small RNA library construction and sequencing

**Figure 2:**
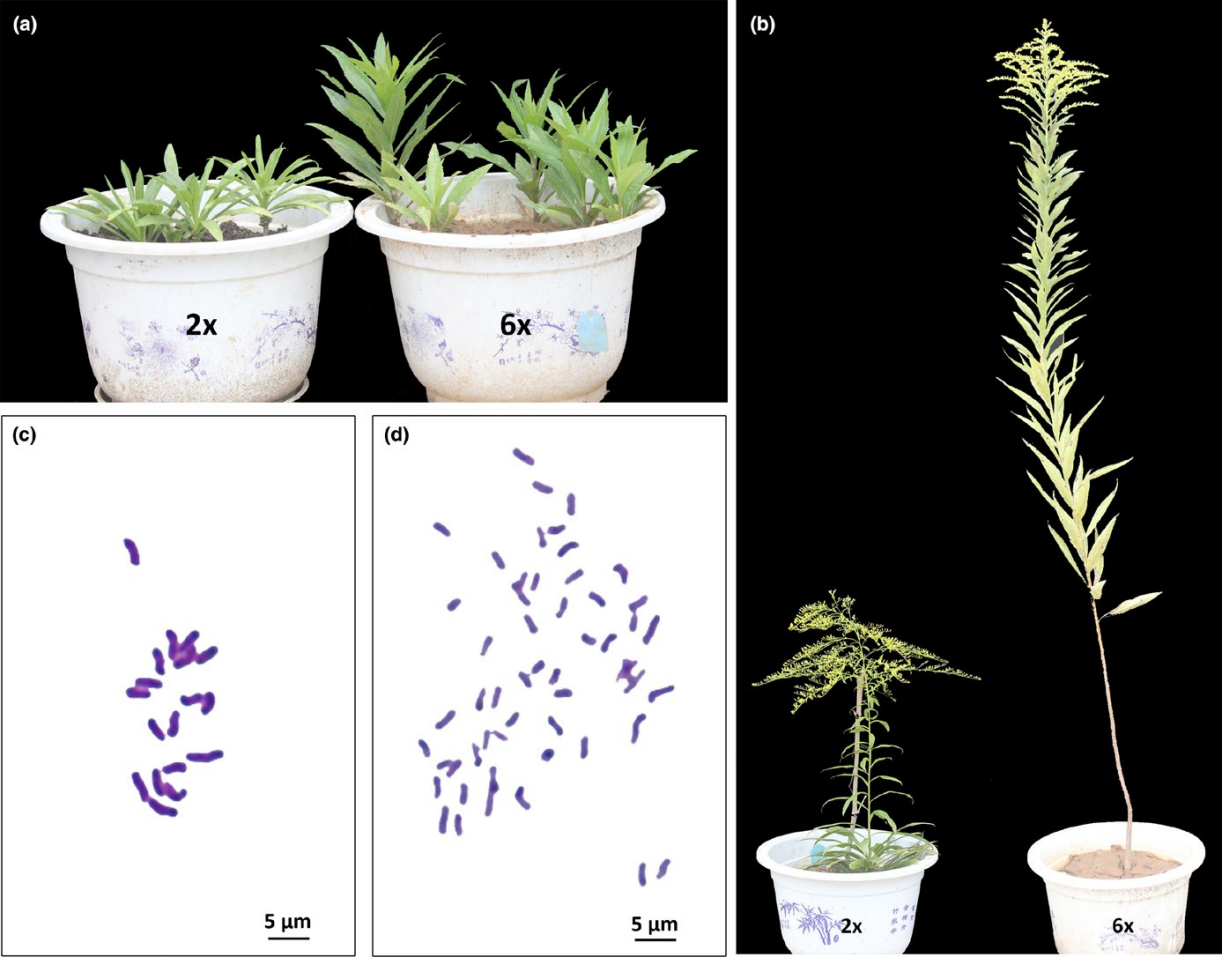
Morphological and cytological divergences between diploid (sampled from CG population, 2*n* = 2*x* = 18) and hexaploid cytotypes (HS 3 population, 2*n* = 6*x* = 54) of *S. canadensis*. Plant morphology of diploid and hexaploid cytotypes in vegetative stage (a) and reproductive stage (b). Chromosome numbers of diploid (c) and hexaploid cytotypes (d). Population names follow Table 1

Based on chromosome survey on the above 43 populations, diploid (population code: CG) and hexaploid cytotypes (HS 3) were selected as the experimental materials for comparison to investigate gene and miRNA expression profiling in this work (Figure 2a,b). Leaves were collected from three independent comparable potted‐seedlings creating three biological replicates for diploid (D) and hexaploid cytotypes (H). The top three to four fully expanded leaves were gently removed in the morning, covered by aluminum foil, frozen in liquid nitrogen immediately, and subsequently stored at −80°C until total RNA extraction. The cDNA and small RNA library were constructed following the methods provided by Beijing Genomics Institute (BGI, Shenzhen, China) (Supporting Information Appendix S1).

### 2.4 De novo assembly and unigene annotation

Raw reads were filtered by removing those reads that contained adaptors, unknown nucleotides (more than 5%), and low‐quality bases (more than 20% of the bases with a quality score less than 15). De novo assembly of all processed reads was performed by Trinity (version: v2.0.6, Grabherr et al., 2011), with parameters set as follows: ‐min_contig_length 200; ‐CPU 8; ‐min_kmer_cov_4; ‐min_glue_4; ‐bfly_opts'‐ V5; ‐edge‐thr=0.1; and ‐stderr'. Then, the constructed transcripts from the Inchworm, Chrysalis, and Butterfly modules of Trinity were further clustered into nonredundant unigenes by using TGICL (version: v2.0.6, Pertea et al., 2003) to eliminate the redundant Trinity‐generated transcripts, with parameters set as follows: ‐I40‐c10‐v25‐O'‐repeat_stringency 0.95‐minmatch 35‐minscore35'. To construct a uniform transcriptome reference, all assembled unigenes from six samples of two cytotypes of *S. canadensis *were pooled together and further clustered to generate “All‐Unigene” for subsequent assembly evaluation, unigene annotation, and expression analysis. The “All‐Unigene” sequences were aligned by BLASTx to a series of protein databases to gain unigene annotation. See Supporting Information Appendix S1 for more unigene annotation details.

### 2.5 Unigene quantification and differentially expressed unigene (DEU) analysis

The Bowtie2 program (version: v2.2.5, Langmead & Salzberg, 2012) was used to map clean reads from each sample to assemble “All‐Unigene” with the following parameters: ‐q; ‐phred 64; ‐sensitive; ‐dpad 0; ‐gbar 99999999; ‐mp 1,1; ‐np 1; ‐score‐minL,0, ‐0.1‐I1‐X 1000; ‐no‐mixed; ‐no‐discordant; ‐p 1‐k 200, and RSEM (version: v1.2.12, Li & Dewey, 2011) was applied to calculate the read counts mapped to each unigene with the default parameter. Then, fragments per kilobase of transcript per million fragments mapped (FPKM) was applied to normalize the expression value. Differential gene expression analysis was performed using the DESeq2 R package as described by Love, Huber, and Anders (2014) for comparisons between diploid and hexaploid cytotypes with three biological replicates. An absolute value of log_2_fold‐change ≥2 and an adjusted *p*‐value <0.001 was set as the threshold to identify DEUs. Following this, identified DEUs were subjected to GO and KEGG analyses. The regulated unigenes were assigned GO terms by the Blast2‐GO program (version: v2.5.0, Conesa et al., 2005), and their enrichment was performed for testing over‐represented GO categories using the GOseq R package with a corrected *p‐*value (FDR analog) setting of ≤0.05. DEUs were further assigned KO (KEGG Orthology) numbers using the KEGG database, and their enrichment was performed as mentioned for GO.

### 2.6 Transcription factor (TF)‐encoding gene prediction

To identify putative TF candidate genes, getorf (version: EMBOSS: 6.5.7.0, Rice, Longden, & Bleasby, 2000) was used to find and extract open reading frames (ORFs) from all assembled unigene sequences with the minimum size parameter set as 150, and then the sequences of ORFs were searched against the plant transcription factor database (PlnTFDB; version: 3.0) using hmmsearch (version: 3.0, Mistry, Finn, Eddy, Bateman, & Punta, 2013) with the default parameters.

### 2.7 miRNA identification and differentially expressed miRNA (DEM) analysis

Raw reads were filtered by removing low‐quality contaminated reads as well as adaptor sequences, and then generated clean reads in the range of 18–30 nt were chosen for mapping to the *S. canadensis* mRNA transcriptome by SOAP with default settings. Subsequently, sequences with a perfect match were compared to Rfam 11.0 and NCBI GenBank databases to eliminate noncoding RNAs, including rRNA, scRNA, snRNA, snoRNA, tRNA, and repeats. Given that sequences from *S. canadensis* were not included in miRBase, the remaining unique reads were searched against currently annotated plant miRNAs (Viridiplantae) available in the miRBase 22.0 database using the BLASTn program to identify the known miRNAs. Transcripts per million was used to normalize the read count of each identified miRNA based on the following formula: Normalized expression = Actual miRNA count × 10^6^/Total count of clean reads. After normalization, differential expression analysis of miRNA was performed using DEGseq as described by Wang, Feng, Wang, Wang, and Zhang (2010). An absolute value of log_2_fold‐change ≥1 and a *q*‐value <0.001 was set as the threshold to identify DEMs.

### 2.8 Prediction of miRNA targets

To predict the potential genes targeted by miRNAs, the Targetfinder (version: 1.5, Fahlgren & Carrington, 2010) in combination with psRobot (version: 1.2, Wu, Ma, Chen, Wang, & Wang, 2012) software was applied to predict as many miRNA targets as possible from the assembled *S. canadensis* unigene set (116,801 “All‐Unigene”) with default parameters. Additionally, the expression level of predicted miRNA targets was taken from the inventory of assembled “All‐Unigene.” GO terms were also evaluated using a similar method.

### 2.9 Visualization of miRNA‐target interaction network

To unravel complex links between candidate miRNAs and unigenes, we proposed a strategy that integrated expression data of DEMs and DEUs to visualize the miRNA‐target interaction network and further discover key miRNAs. Here, we defined coherent miRNA targets as those presenting opposite expression patterns compared with those of the miRNAs, showing that the expression of unigenes was negatively correlated with that of miRNAs (Ye, Wang, & Wang, 2016). To construct the miRNA‐target interaction network, three separate steps were performed. First, DEMs and DEUs were screened following the method mentioned above. Second, predicted targets of up‐regulated miRNAs (down‐regulated miRNAs) overlapped with identified down‐regulated unigenes (up‐regulated unigenes) to obtain coherent miRNA targets. Finally, acquired coherent miRNA targets and DEMs were subjected to visualization of the miRNA‐target interaction network by Cytoscape.

### 2.10 Candidate unigene and miRNA validation via qRT‐PCR

Eighteen promising candidate unigenes and six miRNAs observed to be differentially expressed were chosen for qRT‐PCR to validate the reliability of RNA‐seq and sRNA‐seq results with the following selection criteria: (a) up‐ or down‐regulated unigenes discussed in this paper (i.e., *Expansin*, *ARGOS*); and (b) miRNA‐target interaction pairs that were negatively correlated in expression levels. qRT‐PCR was implemented in triplicate on an ABI Step One Plus Real‐Time PCR System (Applied Biosystems) with unigene‐ and miRNA‐specific sense and anti‐sense primer (Table A1 in Appendix, Supporting Information Appendix S1). A homolog of *GAPDH *(Unigene25510_All) was co‐amplified to normalize the expression values of unigenes and miRNAs in each sample using the double‐standard curve method.

## 3 RESULTS

### 3.1 Gene expression profiling in diploid and hexaploid cytotypes of *S. canadensis*

The inspection of chromosome numbers revealed that two cytotypes were ascertained among the 449 individuals of *S. canadensis* examined. For the cultivated population, all individuals were observed to be diploid cytotypes with a chromosome number of 2*n* = 2*x* = 18 (Figure 2c). For the invasive populations, all individuals were observed to be hexaploid cytotypes with a chromosome number of 2*n* = 6*x* = 54 (Table 1; Figure 2d). However, tetraploid cytotypes with a chromosome number of 2*n* = 4*x* = 36 or mixed‐cytotypes reported by Li (2011) were not found in the current work.

**Figure A2:**
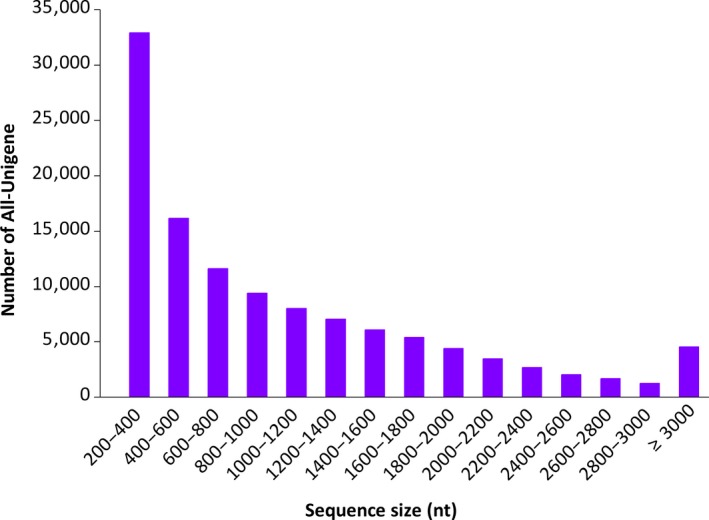
Length distribution of the assembled “All‐Unigene”

To explore key candidate genes behind the invasiveness differences in diploid and hexaploid cytotypes, we generated the first transcriptomic profile of *S. canadensis*. A total of 334.79 million (M) raw reads were produced and subjected to Seq‐QC collating, which resulted in 289.45 M (86%) clean reads with Q20 values ranging from 98.88% to 98.94%. Then, clean reads from six libraries were de novo assembled separately into unigenes by Trinity. These assembled unigenes were pooled together and further clustered into a reference transcriptome (116,801 “All‐Unigene”) with an average length of 1,056 bp, a N50 value of 1,610 bp, and a GC content of 39.20% (Table A2 in Appendix). These numbers are comparable to those generated in other polyploid studies (e.g., Vigna et al., 2016; Zhou et al., 2015) and imply a high‐quality assembly. Furthermore, we also found that the length distribution of the assembled “All‐Unigene” ranged from 224 to 23,608 bp with a total length of 123,376,557 bp, of which 32,942 (28.20%) unigenes ranged from 300 to 500 bp, 32,696 (27.99%) unigenes ranged from 500 to 1,000 bp, 33,468 (28.65%) unigenes ranged from 1,000 to 2,000 bp, and 17,695 (15.15%) unigenes had lengths longer than 2,000 bp (Figure A2 in Appendix).

**Figure A3:**
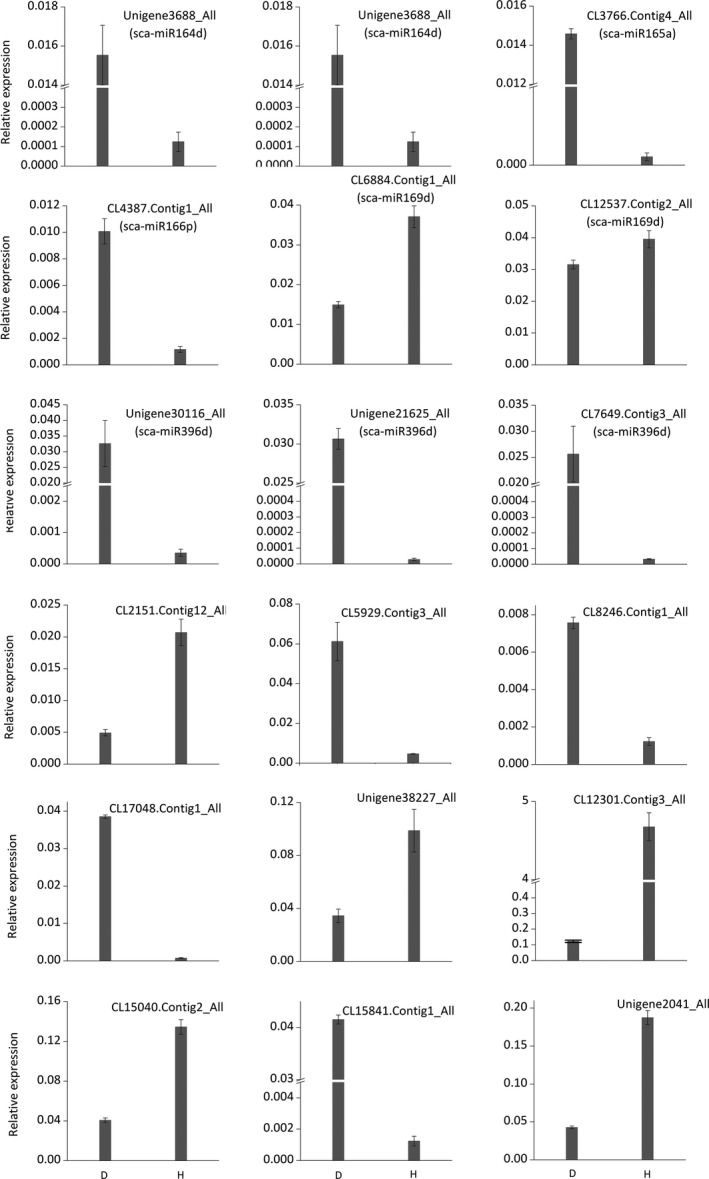
Quantitative qRT‐PCR analysis data of selected ploidy‐responsive 18 unigenes in *S. canadensis*. Error bars represent the standard deviations of three qRT‐PCR replicates. Corresponding coherent miRNAs are indicated in brackets

Out of the 116,801 “All‐Unigene” acquired above, expression of 12,428 unigenes was found only in diploid cytotypes, and expression of 19,520 unigenes was observed only in hexaploid cytotypes. These seem to represent a suite of ploidy‐dependent unigenes, which means a specific role of these ploidy‐dependent unigenes in contrasting invasiveness differences. Subsequently, to identify notably changed unigenes, we applied the aforementioned filter criterion and noticed that 12,897 unigenes displayed at least a four‐fold change in expression levels, with the majority of them (6,768 out of 12,897) down‐regulated in hexaploid cytotypes (Supporting Information Table S1). After that, these DEUs were further subjected to investigation of the specific regulated pathways in which they were involved. However, it must be underlined here that our work has revealed novel unigenes whose functions are unknown, which will be the long‐running theme of future research. Furthermore, qRT‐PCR analysis performed for eighteen DEUs confirmed the mRNA changes detected by RNA‐seq (Figure A3 in Appendix).

**Figure 3:**
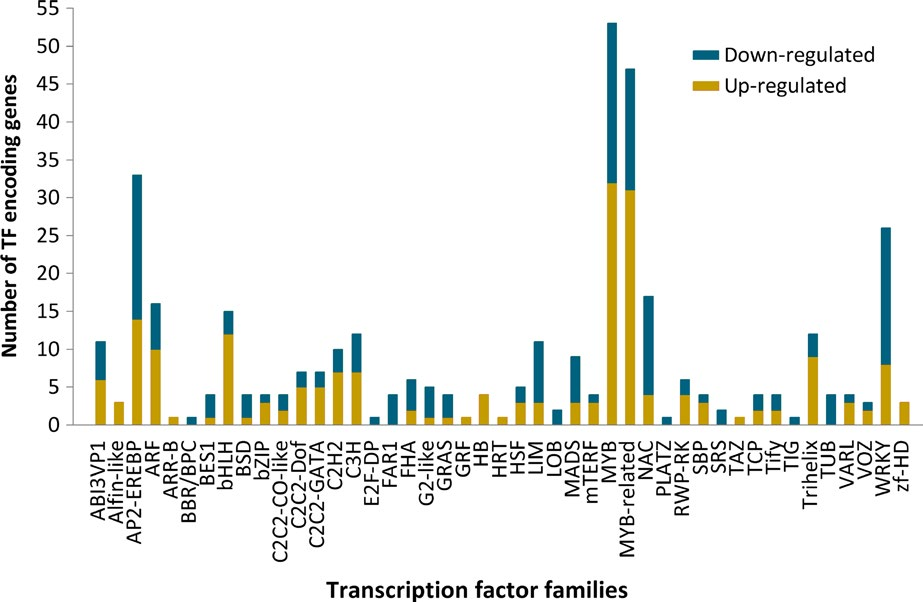
A bar graph representing the differential expression of TF‐encoding genes in diploid and hexaploid cytotypes of *S. canadensis*. Yellow indicates the up‐regulated genes and blue down‐regulated genes in hexaploid cytotypes

Further, the identified 2,644 putative TF‐encoding genes in this work were assigned to 58 TF families, of which MYB members (337) were over‐represented, followed by MYB‐related (270), AP2‐EREBP (211), bHLH (134), and WRKY family members (120). In addition, we discovered 381 TF‐encoding genes that were differentially expressed, with the majority being up‐regulated in hexaploid cytotypes. Notably, among these differentially expressed TF‐encoding genes, almost all the members of the bHLH group were found to be up‐regulated (12/15 genes) in hexaploid cytotypes. In addition, MYB (32/53), MYB‐related (31/47), ARF (10/16) and Trihelix (9/12) members exhibited a similar trend. However, the majority of TF‐encoding genes belonging to the WRKY (18/26) and NAC (13/17) families were down‐regulated in hexaploid cytotypes (Figure 3). These TF genes had differential expression patterns, implying a variety of regulatory modes.

### 3.2 Functional and pathway analysis of ploidy‐responsive unigenes in *S. canadensis*

**Figure A4:**
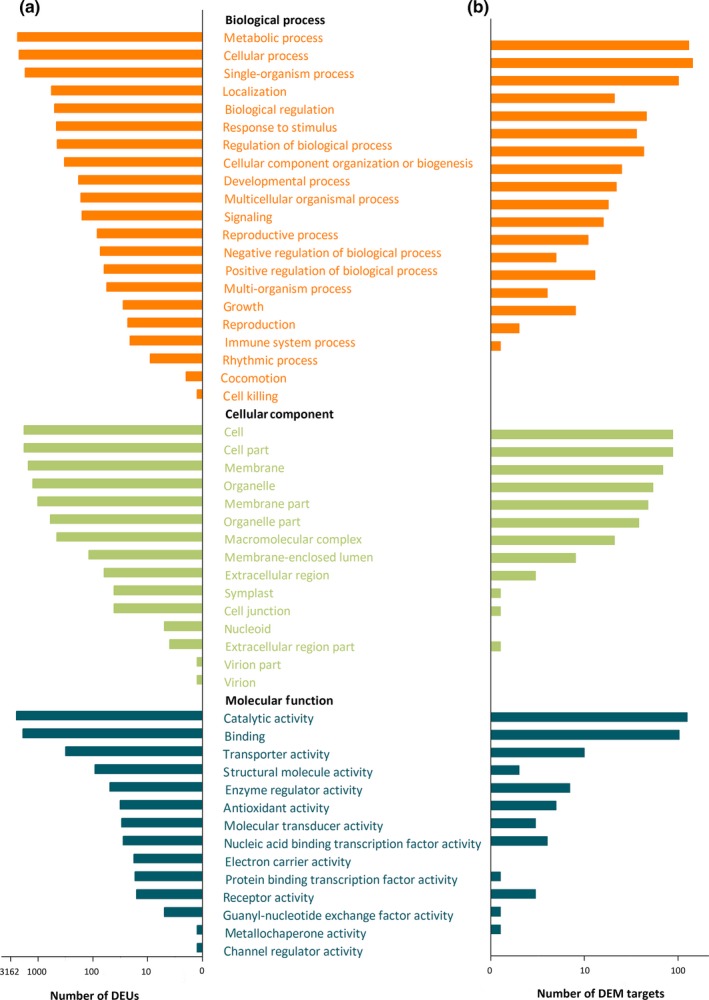
GO‐based functional annotation of differentially expressed unigenes (a) and predicted targets of differentially expressed miRNAs (b)

To better understand the functionality of unigenes differentially expressed in response to ploidy, we mapped the above‐mentioned DEUs to the GO and KEGG databases to perform functional analyses and found that a total of 4,545 (35.24%) unigenes from the 12,897 DEUs were successfully classified into three major functional categories: biological process (3,171), molecular function (3,536), and cellular component (2,764). Then, the three major categories were further assigned to 50 terms (Figure A4a in Appendix), including 21 terms in the biological process, 14 terms in the molecular function, and 15 terms in the cellular component categories. The most abundant GO term related to biological process was “metabolic process” represented by 2,374 DEUs, followed by “cellular process,” “single‐organism process,” “localization,” “biological regulation,” and “response to stimulus” represented by 2,243, 1,741, 568, 501, and 462 DEUs, respectively. In the molecular function category, the two main representative distributions were “catalytic activity” (2,501) and “binding” (1,904). Other GO terms, such as “transporter activity,” “enzyme regulator activity,” “structural molecule activity,” “antioxidant activity,” “receptor activity” and “channel regulator activity,” associated with the biosynthesis of secondary metabolites were also enriched. With respect to the cellular component category, a large proportion of DEUs were clustered in “cell” (1,824), “cell part” (1,808), “membrane” (1,516), and “organelle” (1,264).

**Figure A5:**
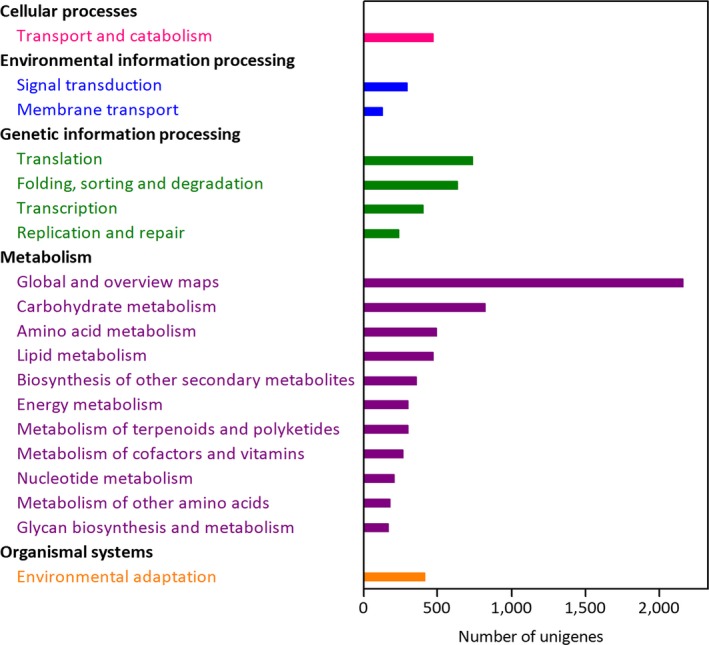
Number of unigenes in each clade of the KEGG pathway maps. The unigenes were assigned 133 KEGG pathways within 19 clades under 5 major categories

In addition, we also found that 8,666 DEUs were assigned to 133 unique KEGG pathways, with 5,705 representing metabolism pathways, 2,006 pathways in genetic information processing, 469 pathways in cellular process, 418 pathways in environmental information processing, and 415 pathways in organismal systems (Figure A5 in Appendix). Notably, there were only two pathways that were significantly over‐represented under “organismal systems,” that is, “circadian rhythm‐plant” and “plant‐pathogen interaction.” The most represented pathway in DEUs was “metabolic pathways,” followed by “biosynthesis of secondary metabolites,” “plant‐pathogen interaction,” “RNA transport,” and “spliceosome.” Subsequently, the hypergeometric distribution was calculated to identify significantly enriched pathways in which DEUs were involved. A total of eight pathways associated with metabolism were significantly enriched, with a *Q* value ≤0.05 (Table A3 in Appendix). It was conspicuous that unigenes related to “metabolic pathways (Pathway ID: ko01100)” were significantly enriched among the DEUs, implying that they may operate in the metabolic adaptation mechanism of hexaploid cytotypes. Additionally, unigenes for carbohydrate metabolism of “pentose and glucuronate interconversions (Pathway ID: ko00040)” were enriched. Moreover, unigenes for lipid metabolism of “fatty acid degradation (Pathway ID: ko00071)” were enriched. Additionally, DEUs involved in the metabolism of terpenoids and polyketides, particularly “carotenoid biosynthesis (Pathway ID: ko00906),” and “sesquiterpenoid and triterpenoid biosynthesis (Pathway ID: ko00909)” were enriched. Finally, “biosynthesis of secondary metabolites (Pathway ID: ko01110),” “isoflavonoid biosynthesis (Pathway ID: ko00943),” and “flavone and flavonol biosynthesis (Pathway ID: ko00944)” were enriched, signifying considerable modulation of unigenes responsible for the regulation of plant secondary metabolites.

### 3.3 miRNA expression profiling in diploid and hexaploid cytotypes of *S. canadensis*

**Figure A6:**
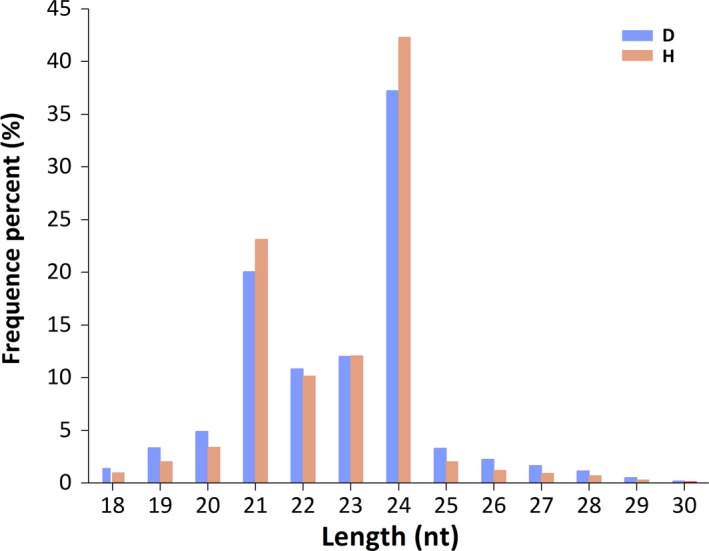
Size distribution of sRNAs in diploid and hexaploid cytotypes of *S. canadensis*

A total of 179.9 M 50‐base pair (bp) single‐end raw reads were produced and subjected to Seq‐QC collating, which resulted in 166.6 M (92.6%) clean reads with lengths ranging from 18 to 30 nt (Table A4 in Appendix). The sRNA length distribution in six libraries showed that the majority of reads were distributed between 20 and 24 nt in length, which corresponds to the size from Dicer‐like digestion products. In addition, the most abundant sequence in all six libraries was 24 nt sRNA (average 37.24% vs. 42.31% in D vs. H), followed by 21 nt sRNA (average 20.06% vs. 23.11% in D vs. H) (Figure A6 in Appendix), which was in agreement with the typical size distribution of sRNAs reported in other plant species, such as Arabidopsis (Rajagopalan, Vaucheret, Trejo, & Bartel, 2006), *Oryza sativa* (Morin et al., 2008), and *Citrus trifoliata* (Song et al., 2010).

We identified 186 miRNAs belonging to 44 miRNA families in two cytotypes of *S. canadensis* and found that the identified families included a changing count of miRNA members (Supporting Information Table S2). Among the detected miRNAs, the miR166 family possessed the largest number of members, with 26 members that were discriminated by the divergences in nucleotide sequences, followed by miR171, miR167, miR168, miR396, miR156, miR169, miR159, miR319, miR164, miR393, and miR160 families, with 14, 12, 11, 11, 10, 10, 8, 8, 6, 6, and 5 members, respectively. miR398, miR399, and miR858 included four members, and miR390, miR395, and miR403 included three members. Of the remaining 26 miRNA families, 12 families, such as miR157, miR161, and miR162 families, comprised two members, and 14 miRNA families were represented only by a single member each.

**Figure A7:**
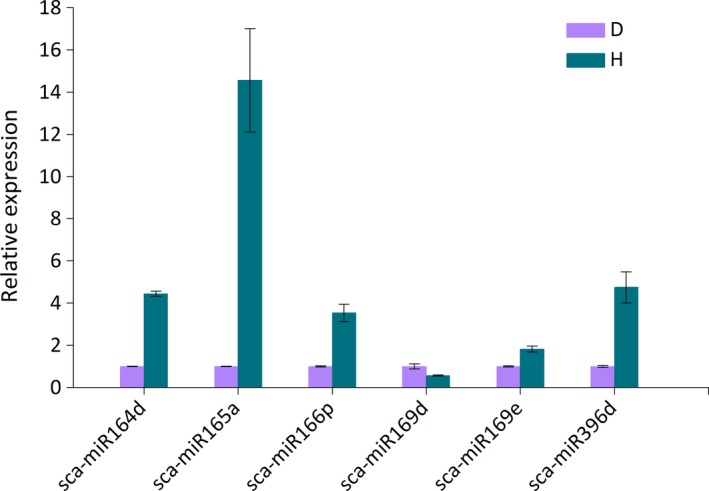
Real‐time qRT‐PCR analyses of miRNAs in diploid and hexaploid cytotypes of *S. canadensis*. The miRNA levels in diploid cytotypes were arbitrarily set as 1. Error bars represent the standard deviations of three qRT‐PCR replicates

**Figure A8:**
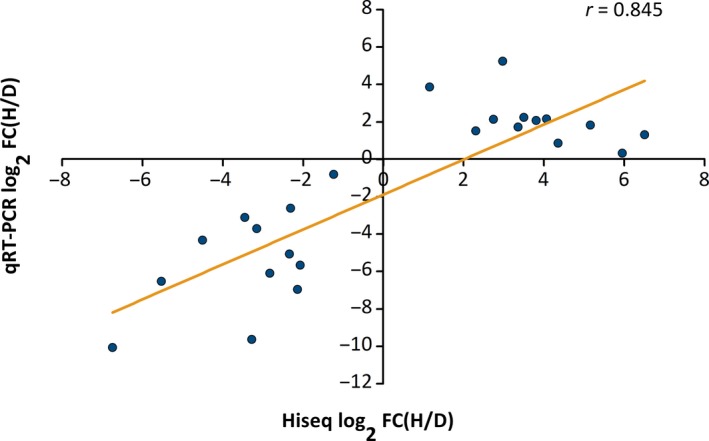
Pearson correlation scatter plot of comparisons of log_2_fold‐change in expression obtained by HiSeq and qRT‐PCR data for unigenes and miRNAs. “*r*” represents the Pearson correlation coefficient

A further analysis showed that 59 miRNAs were differentially expressed, of which 38 miRNAs were up‐regulated and 21 miRNAs were down‐regulated in hexaploid cytotypes relative to their diploids. Among the DEMs, sca‐miR395c, sca‐miR8155, and sca‐miR6173 were markedly down‐regulated with log_2_fold‐change values of −3.23 (*q* = 1.53e−42), −3.15 (*q* = 1.40e−04) and −2.45 (*q* = 6.35e−11), respectively, and sca‐miR166p, sca‐miR528, and sca‐miR396a were markedly up‐regulated with log_2_fold‐change values of 5.16 (*q* = 7.39e−12), 5.13 (*q* = 9.36e−07), and 5.04 (*q* = 5.30e−11), respectively. Notably, for the miR160 and miR169 family, sca‐miR160e, sca‐miR169b, sca‐miR169e, sca‐miR169f, sca‐miR169g, and sca‐miR169h were up‐regulated specifically in hexaploid cytotypes, while sca‐miR160b and sca‐miR169d were up‐regulated specifically in diploid cytotypes. These observations suggested that different members from the same miRNA family had different regulatory modes, probably associated with the cooperative and redundant regulation activity of miRNAs. qRT‐PCR analysis performed for six DEMs confirmed the miRNA changes detected by sRNA‐seq (Figure A7 in Appendix). In addition, correlation between qRT‐PCR results and sequencing results were also calculated. We acquired a significant Pearson “*r*” close to 0.85 (*p* < 0.001) (Figure A8 in Appendix), which strongly suggested that our transcriptome and sRNA sequencing data were credible.

### 3.4 Unigenes involved in growth‐related pathways are targeted by DEMs

Our analysis revealed 1,801 unigenes from 116,801 assembled *S. canadensis* “All‐Unigene” were predicted as targets of 184 miRNAs, of which 884 putative targets were predicted to be cleaved by 58 DEMs. Moreover, a meticulous inspection of the DEMs and their corresponding targets indicated that (a) miR5139a had the highest target abundance (179), and (b) the genes such as CL10163.Contig1_All, CL13112.Contig1_All, and Unigene2861_All had the highest miRNA abundance (4). To understand in depth the group of unigenes targeted by DEMs, GO functional analysis of the predicted targets was carried out. Under the biological process category of GO classification, unigenes involved in terms such as “cellular process,” “metabolic process,” “single‐organism process,” “response to stimulus,” etc. were abundantly enriched as the targets of DEMs. Under the molecular function category, unigenes displaying “catalytic activity,” “binding,” “transporter activity,” etc. were targeted by miRNAs. Moreover, “cell,” “membrane,” “organelle,” etc. related unigenes were discovered to be clustered into the cellular component category as targets (Figure A4b in Appendix). In further pathway analysis of 884 putative targets, “cutin, suberin and wax biosynthesis (Pathway ID: ko00073),” “protein processing in endoplasmic reticulum (Pathway ID: ko04141),” “plant hormone signal transduction (Pathway ID: ko04075),” “selenocompound metabolism (Pathway ID: ko00450)” and “cysteine and methionine metabolism (Pathway ID: ko00270)” pathways were significantly enriched with a *Q* value ≤0.05. These results suggest that miRNAs were more likely to activate plant primary metabolism and make contributions to the improved vigor shown by hexaploid cytotypes, as it has been noted earlier that hexaploid cytotypes typically exhibited enhanced growth in comparison with diploids.

### 3.5 Integrative analysis of gene and miRNA expression confirms that environmental adaptation‐related unigenes are centrally targeted

**Figure 4:**
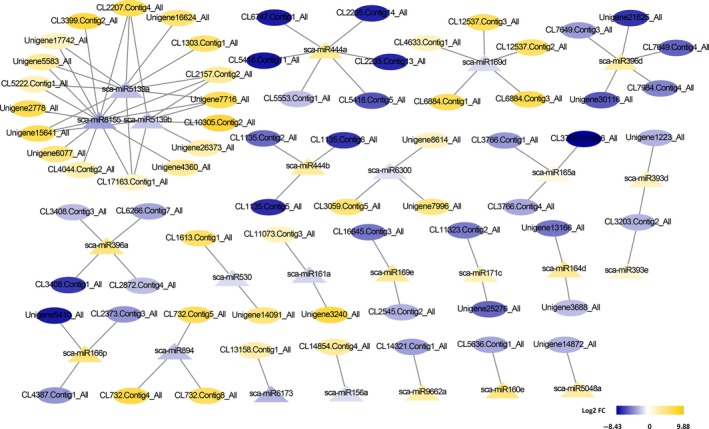
miRNA‐gene interaction network of *S. canadensis*. In this network, oval nodes represented unigenes and triangle nodes represented miRNAs. The negative correlation was denoted by a line. The yellow and blue color mean up‐regulation and down‐regulation and the highest to lowest fold changes are marked from yellow to blue

To detect which biological processes or pathways within a cell were most likely regulated by miRNAs, we integrated overall gene and miRNA expression data to identify miRNA‐target interacting pairs that were negatively correlated in log_2_fold‐change between DEMs and target mRNA expression. As a result, 83 miRNA‐target interacting pairs with the involvement of 24 DEMs and 69 targets were visualized by Cytoscape. For each such pair, we then classified 83 miRNA‐target interacting pairs into two categories depending on the expression patterns of DEMs as either up‐regulated or down‐regulated, respectively, for 47 miRNA‐target pairs involved in 10 down‐regulated miRNAs and 34 up‐regulated targets; or 36 miRNA‐target pairs involved in 14 up‐regulated miRNA and 35 down‐regulated targets (Figure 4). Furthermore, we have also taken note that the coherent miRNA targets included (a) several TFs that were predicted to be targeted by miRNA regulators, for example, sca‐miR164d targets FAR1, sca‐miR530 targets MYB, sca‐miR396a targets Trihelix, and sca‐miR5139a targets VOZ1‐like, suggesting that these miRNAs may operate to enhance the adaptation of hexaploid cytotypes through an integrative miRNA‐TF‐mRNA regulatory network; (b) receptor‐like protein kinases (RLKs) that were predicted to be targets of multiple miRNAs such as sca‐miR161a, sca‐miR5139a, sca‐miR5139b, and sca‐miR8155. This target is an important enzyme gene and functions in regulating plant growth, development, signal transduction, immunity, and stress responses (Sun, Li, Wang, Zhang, & Wu, 2017). Notably, these RLK genes were remarkably up‐regulated in hexaploid cytotypes, suggesting that their regulator miRNAs may play key roles in the environmental adaptation of hexaploid cytotypes; (c) unigenes associated with methylation and ubiquitination processes, such as histone‐lysine *N*‐methyltransferase (CL7649.Contig3_All), ubiquitin‐protein ligase (CL2235.Contig13_All), U‐box domain‐containing protein (CL2207.Contig4_All), and F‐box protein (Unigene1223_All), that were predicted to be targets of sca‐miR396d, sca‐miR444a, sca‐miR393d, and sca‐miR5139a, suggesting that these unigenes may be subjected to miRNA‐mediated DNA methylation and ubiquitination; and (d) two dirigent protein genes (CL6884.Contig1_All and CL6884.Contig3_All) that were predicted to be targets of sca‐miR169d. This target was an unspecific oxidizing enzyme gene for radical formation that functions in lignan biosynthesis, which was previously reported to be an integral regulator of plant secondary metabolism (Effenberger et al., 2015). Notably, these dirigent protein genes were remarkably up‐regulated in hexaploid cytotypes, suggesting that its regulator sca‐miR169d may play key roles in plant secondary metabolism (Table A5 in Appendix).

## 4 DISCUSSION

A large number of works have investigated ecological and evolutionary elements responsible for successful invasion (Hahn et al., 2012; Thébault et al., 2011). However, research into the molecular basis for invasiveness in invasive plants is just getting started. Here, we found 12,897 unigenes and 59 miRNA regulators with divergences in expression between diploid and hexaploid cytotypes. Intriguingly, among them were an over‐representation of unigenes and coherent miRNA targets relevant to metabolism, plant growth and development, and stress responses, implying that these modified genetic and epigenetic attributes may harbor both biochemical and ecological advantages that were beneficial to the successful invasion of hexaploid cytotypes.

### 4.1 Unique gene and miRNA expression characteristics might have contributed to the invasiveness of hexaploid cytotypes

In *Arabidopsis thaliana*, only 0.1% differences in gene expression between diploid and autotetraploid were detected (Yu et al., 2010). In newly synthesized autotetraploid *Paspalum notatum*, 0.6% of genes were differentially expressed compared to its diploid (Martelotto et al., 2005). Similarly, the analysis of 21,081 genes in *Citrus limonia* autotetraploids revealed less than 1.1% differences in comparison with diploids (Allario et al., 2011). In contrast, many researchers have observed a more noticeable transcriptomic divergence between allopolyploids and their parents in several plants (Li et al., 2014; Ye et al., 2016). Remarkably, here we detected >10% transcriptomic differences as a consequence of hexaploid cytotype formation. Two factors may account for this dramatic change. First, *S. canadensis* is a polyploid, and assembling its transcriptome has been exceedingly difficult because it principally comprises highly similar repeats, thereby causing several contigs that often represent nonoverlapping fragments of the same unigene. Second, given that polyploid effects on gene expression might be induced by genome doubling and/or hybridization, we speculate that the expression pattern of hexaploid cytotypes of *S. canadensis* should be that of an allohexaploid. Furthermore, a significant caveat in the interpretation of these results is that we have only sequenced one population per ploidy level, and genetic differentiation among different geographic populations of the same ploidy could also be contributing to gene and miRNA expression differences. Thus, further work is essential to explore the genetic relationship between cytotypes. Furthermore, to test whether the invasiveness difference between plants was reflected in the changes of their gene expression patterns, we examined evidence for successful invasion in introduced populations across multiple invasive plants. Here, common‐garden studies comparing native and introduced populations of *Cirsium arvense*, *Centaurea diffusa*, and *Mikania micrantha*, as well as comparisons between *S. canadensis* and invasive taxa of the Asteraceae, have been performed, and obvious similarities have emerged (Guggisberg, Lai, Huang, & Rieseberg, 2013; Guo et al., 2018; Hodgins et al., 2015; Turner, Nurkowski, & Rieseberg, 2017). In these studies, introduced populations notably differ from their native populations with regard to stress response. In line with this observation, the significantly different regulation of stress response genes, such as receptor‐like proteins, is of particular interest between introduced and native populations because these genes mediate plant cellular defense pathways. Similar, several stress response genes associated with secondary metabolism, such as the cytochrome P450 gene family, were also found to be significantly expressed in the present work. Therefore, it is reasonable to speculate that these stress response genes might have crucial functions in invasive characteristics. However, we also noticed that genes involved in photosynthesis were exclusively enriched in *M. micrantha *(Guo et al., 2018). Hence, it seems that the pattern of gene expression across different invasive plants is dependent on a specific plant, and thus, it is difficult to generalize a rule of gene expression during invasion.

Likewise, the relative amount of miRNAs was higher in a derived hexaploid wheat (BBAADD) than in the parental tetraploid *Triticum turgidum *ssp. *durum* (BBAA) and diploid *Aegilops tauschii *(DD) (Kenan‐Eichler et al., 2011). Analogously, the number of miRNA or miRNA families in cultivated allotetraploid cotton *G. hirsutum* (AADD) was markedly greater than those in its two diploid ancestors, *G. raimondii *(DD), and *G. arboreum *(AA) (Xie & Zhang, 2015). Ghani et al. (2014) also reported that the percentages and expression levels of miRNAs increased in allodiploid (AB) and allotetraploid (AABB) relative to the parents *Brassica rapa *(AA) and *Brassica nigra *(BB). In the present work, the number and expression levels of miRNAs in hexaploid cytotypes were greater than those in their diploids, which was consistent with the findings of the above‐mentioned studies. These results suggest that an increase in ploidy was generally coupled with an obvious increase in the percentages and expression levels of miRNAs.

### 4.2 Several regulatory mechanisms seem to operate gene expression properly in hexaploid cytotypes

How does hexaploid cytotypes regulate the differential expression of unigenes? Several mechanisms could be associated with this regulation. miRNAs work as regulators for controlling target‐gene expression, thereby affecting a variety of aspects of phenotype, growth, development, and stress response (Ha et al., 2009). Here, we showed a subset of key candidate miRNA regulators within diploid and hexaploid cytotypes and used these DEMs to predict putative targets using two different target‐prediction software. To shed light on the regulatory action of these DEMs, we compared these predicted targets with DEUs based on GO functional classification (Figure A4a,b in Appendix) and found that biological processes were highly likely to be regulated by miRNAs, such as (a) a considerable proportion of the enriched unigenes were clustered in “biological process”; (b) processes such as “metabolic” and “cellular” were abundantly enriched; and (c) “single‐organism,” “localization,” “biological regulation,” and “response to stimulus” were also adequately reflected. Such observations suggested that unigenes described in the above‐mentioned terms were most likely targeted by miRNAs. However, unigenes associated with “cell killing,” “locomotion,” and “rhythmic process” were enriched only in DEUs, implying that although unigenes associated with the foregoing processes were differentially expressed, this regulation of gene expression cannot be attributed to the miRNA‐induced cleavage of targets. In contrast, no term was only enriched under the same category for the targets of DEMs. These observations suggested that few specific biological processes were regulated by miRNAs. Similarly, “channel regulator activity” and “electron carrier activity” enriched in the “molecular function” category were only amid DEUs. In addition, under the category of “cellular component,” unigenes related to terms “cell,” “membrane” and “organelle” were overwhelming in this comparison, whereas no unigene associated with terms “nucleoid,” “virion,” and “virion part” was enriched among the targets of DEMs, indicating that these unigenes are closely regulated at the transcriptional level and may not be prominently influenced by miRNA‐induced gene silencing.

In addition to miRNAs, other accessional regulation manners, such as DNA methylation, may also function to regulate gene expression. There is impressive evidence that an allopolyploid's intergenomic interactions between two divergent genomes were projected to incur DNA methylation changes, eventually causing the differential expression of genes, which can potentially lead to profound phenotypic consequences (Chen, 2007; Salmon & Ainouche, 2010). DNA methylation changes between an allopolyploid and its parents have been very well reported. For instance, in *Spartina* allopolyploids, a high level of epigenetic regulation might explain the morphological plasticity and its larger ecological amplitude (Salmon, Ainouche, & Wendel, 2005). Additionally, Madlung et al. (2002) reported that changes in DNA methylation would result in the development of altered morphologies in synthetic allotetraploids. Although DNA methylation alterations are principally observed in allopolyploids, activation, or repression of gene expression has also been shown to correlate with DNA methylation variation in autopolyploid Arabidopsis (Yu et al., 2010) and *Cymbopogon *(Lavania et al., 2012). In the present work, a large number of DEUs related to epigenetic regulation were investigated in two cytotypes of *S. canadensis *(Supporting Information Table S3). In particular, transcriptome analysis defined eleven unigenes (annotated as DNA (cytosine‐5)‐methyltransferase1 gene, for example, CL12526.Contig3_All, CL5231.Contig1_All, and CL5231.Contig2_All) that displayed striking changes in gene expression. Interestingly, almost all the DNA (cytosine‐5)‐methyltransferase1 genes were found to be significantly down‐regulated (10/11 genes) in hexaploid cytotypes, and these observations should be further investigated because such genes could potentially participate in the maintenance of CG methylation. Additionally, to answer developmental and environmental alterations, chromatin composed of DNA and histones in eukaryotic cell nuclei is modulated by several histone modifications. Among these modifications, histone demethylation regulates gene expression mainly by demethylating histone lysine residues (Shi & Tsukada, 2013). Recent studies have identified Jumonji (JmJ) proteins to be involved in histone demethylation and closely related to the reproductive process. The loss‐of‐function mutations of the rice gene *JmJ706* resulted in spikelet development defects (Sun & Zhou, 2008). Here, a total of eight DEUs (e.g., CL1566.Contig6_All, CL1566.Contig9_All, and CL1566.Contig12_All) were annotated as *JmJ* genes, and all of them were up‐regulated in hexaploid cytotypes, which might partly suggest that the JmJ histone demethylase unigenes may alter the expression of a large number of target genes and contribute to the variation in physiology, biochemistry and phenotype between diploid and hexaploid cytotypes. However, further study is needed. Taken together, these data clearly state that complicated and overlapping gene expression regulatory mechanisms may have evolved in hexaploid cytotypes to guarantee suitable transcriptional control in response to environmental stimuli.

### 4.3 Potential roles of transcriptional alterations in the successful invasion of hexaploid cytotypes

Polyploids play recognized roles in driving organ size and growth of plants. The leaf is the main photosynthetic organ, and its size strongly affects the energy capture, photosynthetic capacity, and physiological activities of plants (Baute et al., 2017; Niinemets, Portsmuth, & Tobias, 2006). The coordination of cell proliferation and expansion is a crucial determinant that serves a critical function in precisely controlling leaf size and growth caused by cell ploidy (Baute et al., 2017; Marshall et al., 2012; Sugiyama, 2005), which have been previously suggested to be regulated by a number of genes encoding transcription factors, modification proteins, plant hormones, and cell wall protein. The Growth‐Regulating‐Factor (GRF) protein, a plant‐specific transcription factor, has been confirmed to affect leaf growth by positively regulating cell proliferation, cell expansion, and adaxial‐abaxial patterning (Omidbakhshfard, Proost, Fujikura, & Mueller‐Roeber, 2015). In addition, the GRF protein has also been shown to perform transcription regulation functions by interacting with GRF‐Interacting Factor (GIF) protein (Debernardi et al., 2014). In this work, five unigenes (unigene52239_All, CL15674.Contig2_All, CL8349.Contig2_All, CL1635.Contig4_All, and CL1635.Contig1_All) annotated as *GRF *and two unigenes (Unigene15938_All and CL6058.Contig2_All) annotated as *GIF* were found to be differentially expressed and may form functional complexes potentially implicated in leaf size and growth. Furthermore, other transcription factors, such as the TCP transcription factor (e.g., CL7816.Contig2_All, CL9341.Contig3_All), were also identified as regulators of leaf size. Except for TFs, regulatory proteins act as important regulators of leaf size and growth by influencing cell proliferation. EBP1, an ortholog of ErbB3‐binding protein from humans, regulates leaf size and growth by cell proliferation. Some studies highlight that the expression of *EBP1* correlates with plant organ size, growth, and stress tolerance (Cao et al., 2009; Horváth et al., 2006). In the present work, one ortholog of *EBP1*, CL16506.Contig4_All, was found to be significantly up‐regulated in hexaploid cytotypes, which may be responsible for the larger leaves, faster growth, and better stress resistance of hexaploid cytotypes. Similarly, F‐box proteins, which are members of regulatory protein families that affects leaf size (Baute et al., 2017), are abundantly expressed. Moreover, earlier studies showed that auxin mediated the expression of multiple genes (e.g., *ARGOS* and *ARF*) to affect plant organ size and growth (Schruff et al., 2006; Wang, Zhou, Xu, & Gao, 2010). *Auxin‐Regulated Gene involved in Organ Size* (*ARGOS*), a gene deeply induced by auxin, participate in organ size regulation. Wang, Zhou, et al. (2010) also pointed out that overexpression of a Chinese cabbage (*Brassica rapa*) *BrARGOS* gene in Arabidopsis elevates the size of plant organs. In this work, an ortholog of *BrARGOS*, CL15040.Contig2_All was detected, and the up‐regulated expression may have similar functions in the organ giantism observed in hexaploid cytotypes. Auxin Response Factor (ARF), a transcription factor, functions in plant size, growth, and stress adaptation by transcriptionally activating and repressing the expression of auxin response genes (Zhao, Zhang, Ma, & Wang, 2016). Here, 16 ARF encoding genes were found to be differentially expressed, which might contribute to invasiveness differences between diploid and hexaploid cytotypes. Lastly, abundant studies have shown correlations between expansin gene expression and cell wall remodeling, growth and stress response, and phenotype changes in plants (Goh, Sloan, Malinowski, & Fleming, 2014; Lee & Choi, 2005; Li et al., 2013), which supports the roles for expansin as an important cell wall protein in plant cell wall modification, growth promotion, and stress tolerance. As expected, the overexpression of expansin genes has remodeled leaf structure, which confers them enhanced tolerance to abiotic stresses (Cho & Cosgrove, 2000; Kwon et al., 2008). In the present work, thirteen unigenes encoding expansins were differentially expressed, and eleven of them were more highly expressed in hexaploid cytotypes of *S. canadensis* than in diploid cytotypes. These results suggested that the activation of expansins may be a rapid growth and adaptation mechanism of hexaploid cytotypes in novel heterogeneous environments.

Furthermore, polyploids can also profoundly affect plant metabolism qualitatively and quantitatively, furnishing the chance for increased metabolic activity through transcriptional divergence, which eventually results in alterations in the levels of secondary metabolites (Fasano et al., 2016). There are multiple studies on the induction of polyploids to promote the production of specific secondary metabolites. For instance, autotetraploids of *Catharanthus roseus* produced more vindoline, catharanthine, and vinblastine than their diploids (Xing et al., 2011). *Echinacea purpurea* autotetraploids showed that the induction of polyploids resulted in higher caffeic acid derivatives and alkamides (Xu et al., 2014). Evidence of the influence of polyploids on chemical profiles has also been recorded in allopolyploids. Banyai et al. (2010) reported that allotetraploid *Artemisia annua* produced more terpenoids or triterpene‐type compounds than diploids. Supporting the role of plant secondary metabolism in polyploid‐mediated invasiveness differences “biosynthesis of secondary metabolites (Pathway ID: ko01110)” was found to be the most significantly enriched pathway with a *Q* value far below 0.05 in the pathway enrichment analysis of DEUs in the present work. Taking the above into account, we propose that polyploids are more likely to remodel the transcriptome and metabolome in hexaploid cytotypes, resulting in ploidy‐specific metabolic adaptation. Moreover, a marked number of DEUs encoding enzymes related to plant metabolism were observed, which further supports this plausible explanation. The synthesis of secondary metabolites primarily contains the oxidation, reduction, and cyclization steps, in which unigenes encoding enzymes of cytochrome P450 (CYPs) and uridine diphosphate glucuronosyl transferases (UGTs) play crucial roles in catalyzing these reactions (Zhang et al., 2016). Based on the functional annotation of DEUs, a total of 120 core enzyme unigenes encoding CYPs were differentially expressed (Table A6 in Appendix). In addition, CYPs are one of the largest superfamilies of enzyme proteins (Darabi, Seddigh, & Abarshahr, 2017). A large number of CYPs are involved in a wide range of biosynthetic reactions and biochemical pathways, leading to the synthesis of UV protectants (flavonoids and anthocyanins), defensive compounds (isoflavonoids, phytoalexins, hydroxamic acids, and terpenes), fatty acids, hormones (gibberellins and brassinosteroids), signaling molecules (oxylipins, salicylic acid, and jasmonic acid), accessory pigments (carotenoids), and structural polymers such as lignins (Darabi et al., 2017; Schuler & Werck‐Reichhart, 2003). In the present work, many CYP‐related unigenes were identified, such as *CYP93A* (e.g., CL361.Contig6_All, CL1330.Contig7_All, CL16738.Contig1_All), *CYP76B* (e.g., CL3689.Contig2_All, CL1852.Contig2_All), and *CYP71* (e.g., CL6714.Contig2_All, Unigene23159_All, CL15279.Contig1_All), which respectively participated in the biosynthesis of isoflavonoids, flavonoids, and sesquiterpenoids and triterpenoids, which may act as defensive compounds that protect against oxidative damage under abiotic stress. Furthermore, a great deal of UGT genes that participated in flavonoid biosynthesis, such as *UGT73*, *UGT74*, *UGT76*, *UGT83*, *UGT85,* and *UGT89*, were also identified (Table A7 in Appendix). Given these findings, it is attractive to investigate the potential model whereby polyploids impact the metabolome in hexaploid cytotypes of *S. canadensis*.

**Figure 5:**
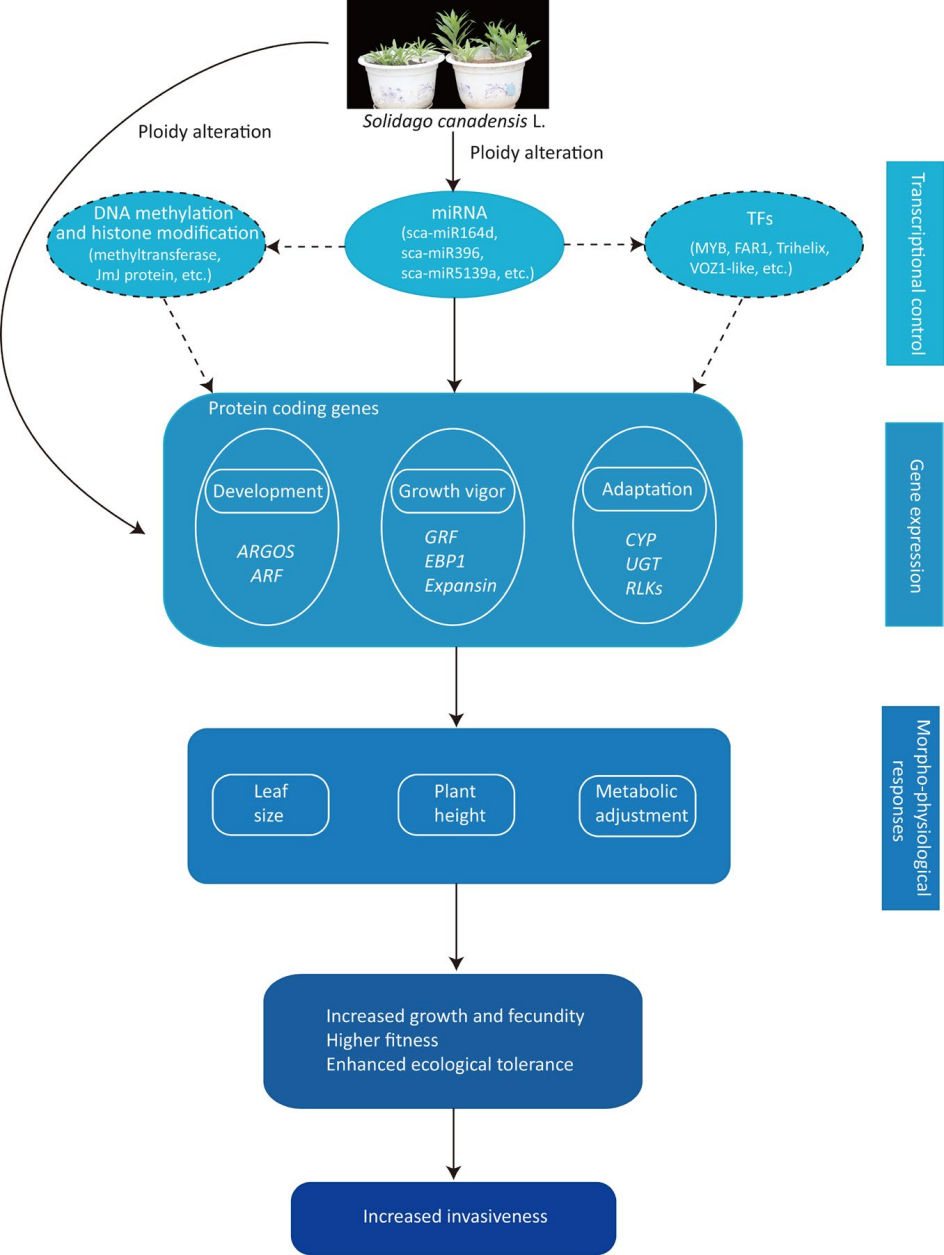
Graphical summary of molecular responses to ploidy alteration in *S. canadensis*. Dotted lines and dashed boxes represent the putative regulations

In conclusion, important candidate unigenes and miRNA regulators that contributed to the successful invasion of hexaploid cytotypes of *S. canadensis *have been investigated in the current work, and we have also further inferred ploidy‐related regulation of DNA methylation as an additional modulatory event that occurs to modulate transcriptome reprogramming to drive invasion success. Furthermore, a model for depicting the events involved in ploidy alteration in *S. canadensis* is summarized in Figure 5. Collectively, this work not only describes which molecular processes and functional pathways are likely vital in the successful invasion of polyploids but also offers a valuable dataset for future functional experiments aiming to determine which of these candidate unigenes and miRNA regulators truly underlie the differences in invasiveness between diploid and hexaploid cytotypes.

## CONFLICT OF INTEREST

None declared.

## AUTHOR CONTRIBUTIONS

C.C.X. collected plants, performed greenhouse experiment, analyzed the collected data, and wrote this manuscript; Y.M.G. participated in data analysis; J.B.W. conceived the research and contributed to data interpretation and revisions of this manuscript.

## Data Availability

The mRNA‐seq and sRNA‐seq data as fastq files from diploid and hexaploid cytotypes of *S. canadensis *were deposited in NCBI SRA database under the accession number SRP152671.

## APPENDIX 1

**Table A1:** Primers designed for qRT‐PCR analysis of unigenes and miRNAs

MiRNA/unigene	Primers		
	RT primer	Sense primer	Anti‐sense primer
sca‐miR164d	GTCGTATCCAGTGCAGGGTCCGAGGTATTCGCACTGGATACGACGCACGT	GCCGCTTGGAGAAGCAGGGC	GTGCAGGGTCCGAGGT
sca‐miR165a	GTCGTATCCAGTGCAGGGTCCGAGGTATTCGCACTGGATACGACGGGGAT	GCCGCTTCGGACCAGGCTTC	GTGCAGGGTCCGAGGT
sca‐miR166p	GTCGTATCCAGTGCAGGGTCCGAGGTATTCGCACTGGATACGACCCTTGA	GCCGCTGGAATGTTGTCTGGT	GTGCAGGGTCCGAGGT
sca‐miR169d	GTCGTATCCAGTGCAGGGTCCGAGGTATTCGCACTGGATACGACAGGCAA	GCCGCTTAGCCAAGGATGAC	GTGCAGGGTCCGAGGT
sca‐miR169e	GTCGTATCCAGTGCAGGGTCCGAGGTATTCGCACTGGATACGACTCGGCA	GCCGCTCAGCCAAGGATGACT	GTGCAGGGTCCGAGGT
sca‐miR396d	GTCGTATCCAGTGCAGGGTCCGAGGTATTCGCACTGGATACGACTTTCCC	GCCGCTGTTCAATAAAGCTGT	GTGCAGGGTCCGAGGT
Unigene3688_All		TGTAAACGCTTCCGTAAATCAG	TGCCACAATAGACAACCCAC
Unigene13166_All		GGATGGAGTAAGCCGAGGAT	GAAGTGACCGTGACAATGACC
CL3766.Contig4_All		TCTGGTTTCCGTATCATTCCT	CCGATGCGATGTCCCTAA
CL4387.Contig1_All		CTGGCTGTCATCACCTTTGG	CCTCTAACTGCTGCTGCGTAT
CL6884.Contig1_All		AGCATACCCAGTAGCAAACCG	TCAAAGATGATTGGAAAGGCAC
CL12537.Contig2_All		TTCAGACATAAGGGAAGGTGGT	ATCCGAAAATGAGATAAGAGGC
Unigene30116_All		ACTGCCGTCTCCGATTTATG	CTGCTTATGTATGCCTCTGTCTTC
Unigene21625_All		AGACAGAGGCATACATAAGCAGG	ACACCCAAAGGGGAGACCA
CL7649.Contig3_All		GAAGACAGAGGCATACATAAGCAG	ACACCCAAAGGGGAGACCA
CL2151.Contig12_All		TCGCTCAGGGTATTCAGGT	CATTTCACGGAAGGGGTT
CL5929.Contig3_All		CTAACTGGAGTATCGCCGTGTC	CGGGTCATTCGCTTCTTTG
CL8246.Contig1_All		CAAGCCCTTCATCCATCTATT	CAAACCCCATTTCCCTCA
CL17048.Contig1_All		GAGTTCAGGGATGTATGGAGGTT	GATGATAGTTGTCCGCACAGATT
Unigene38227_All		CGTAATAAGCAGGTGGCATCA	CAAGCATTTTCCCAGCAGAGT
CL12301.Contig3_All		TGGAAGAACCATCACGAGC	AACACTACGACCCGAACGA
CL15040.Contig2_All		CGACACCGTTAGTCCAAGCA	GACAACAAAGGCGGCATACA
CL15841.Contig1_All		TGTAAGGCAAAGGCACTGGG	CGGCTTCTTCGGCTGTATCA
Unigene2041_All		CGGGGTCTTTACAGGTTACTCG	TGTGCTCCACTCCAGCGTTT
GAPDH		TAAGGTCGTCGCTTGGTA	TCTTCTTCGGATGGGTTC

**Table A2:** Overview of sequencing data of trinity‐assembled *S. canadensis* transcriptome

Category	D1	D2	D3	H1	H2	H3
Raw reads (Mb)	55,530,162	55,530,388	55,530,274	57,163,624	55,530,288	55,511,070
Clean reads (Mb)	48,699,934	48,275,088	48,972,390	48,416,760	48,261,972	46,824,558
Q20 (%)	98.94	98.89	98.89	98.89	98.94	98.88
Unigenes	55,769	56,001	58,471	57,867	58,152	56,528
Mean length	965	949	953	944	904	950
N50	1,458	1,403	1,433	1,449	1,377	1,455
GC (%)	39.67	39.59	39.62	39.80	39.84	39.81

Note

D1‐D3 and H1‐H3 correspond to diploid and hexaploid cytotypes of *S.canadensis*, respectively.

**Table A3:** KEGG pathway enrichment analysis of differentially expressed unigenes

No.	Pathway	DEU number	*Q* value	Pathway ID	Level 1
1	Biosynthesis of secondary metabolites	1,217 (14.04%)	9.11E−06	ko01110	Metabolism
2	Carotenoid biosynthesis	63 (0.73%)	4.40E−05	ko00906	Metabolism
3	Metabolic pathways	2,022 (23.33%)	1.73E−04	ko01100	Metabolism
4	Sesquiterpenoid and triterpenoid biosynthesis	45 (0.52%)	1.61E−02	ko00909	Metabolism
5	Fatty acid degradation	70 (0.81%)	1.79E−02	ko00071	Metabolism
6	Pentose and glucuronate interconversions	141 (1.63%)	4.00E−02	ko00040	Metabolism
7	Isoflavonoid biosynthesis	42 (0.48%)	4.00E−02	ko00943	Metabolism
8	Flavone and flavonol biosynthesis	44 (0.51%)	4.00E−02	ko00944	Metabolism
9	Steroid biosynthesis	49 (0.57%)	6.31E−02	ko00100	Metabolism
10	Nitrogen metabolism	53 (0.61%)	6.31E−02	ko00910	Metabolism
11	Anthocyanin biosynthesis	22 (0.25%)	6.31E−02	ko00942	Metabolism
12	Tryptophan metabolism	61 (0.7%)	6.31E−02	ko00380	Metabolism
13	Amino sugar and nucleotide sugar metabolism	162 (1.87%)	6.48E−02	ko00520	Metabolism
14	Tyrosine metabolism	59 (0.68%)	6.77E−02	ko00350	Metabolism
15	Monobactam biosynthesis	28 (0.32%)	6.77E−02	ko00261	Metabolism
16	Glutathione metabolism	62 (0.72%)	7.37E−02	ko00480	Metabolism
17	Arginine and proline metabolism	72 (0.83%)	9.97E−02	ko00330	Metabolism
18	Lysine biosynthesis	28 (0.32%)	9.97E−02	ko00300	Metabolism
19	Flavonoid biosynthesis	63 (0.73%)	9.97E−02	ko00941	Metabolism
20	Indole alkaloid biosynthesis	11 (0.13%)	1.19E−01	ko00901	Metabolism
21	Ubiquinone and other terpenoid‐quinone biosynthesis	57 (0.66%)	1.24E−01	ko00130	Metabolism
22	Butanoate metabolism	30 (0.35%)	1.28E−01	ko00650	Metabolism
23	Degradation of aromatic compounds	18 (0.21%)	1.30E−01	ko01220	Metabolism
24	Circadian rhythm—plant	85 (0.98%)	1.36E−01	ko04712	Organismal Systems
25	Valine, leucine and isoleucine degradation	80 (0.92%)	1.50E−01	ko00280	Metabolism
26	Vitamin B6 metabolism	22 (0.25%)	1.50E−01	ko00750	Metabolism
27	C5‐Branched dibasic acid metabolism	18 (0.21%)	1.88E−01	ko00660	Metabolism
28	Nonhomologous end‐joining	11 (0.13%)	2.09E−01	ko03450	Genetic Information Processing
29	alpha‐Linolenic acid metabolism	59 (0.68%)	2.09E−01	ko00592	Metabolism
30	Porphyrin and chlorophyll metabolism	62 (0.72%)	2.09E−01	ko00860	Metabolism
31	Arachidonic acid metabolism	32 (0.37%)	2.09E−01	ko00590	Metabolism
32	Fatty acid metabolism	83 (0.96%)	2.09E−01	ko01212	Metabolism
33	Phenylalanine metabolism	45 (0.52%)	2.09E−01	ko00360	Metabolism
34	Stilbenoid, diarylheptanoid and gingerol biosynthesis	63 (0.73%)	2.09E−01	ko00945	Metabolism
35	Ether lipid metabolism	45 (0.52%)	2.14E−01	ko00565	Metabolism
36	Limonene and pinene degradation	55 (0.63%)	2.27E−01	ko00903	Metabolism
37	Brassinosteroid biosynthesis	15 (0.17%)	2.27E−01	ko00905	Metabolism
38	Mismatch repair	131 (1.51%)	2.27E−01	ko03430	Genetic Information Processing
39	Ascorbate and aldarate metabolism	68 (0.78%)	2.29E−01	ko00053	Metabolism
40	Fatty acid biosynthesis	36 (0.42%)	2.73E−01	ko00061	Metabolism
41	Glycolysis/ Gluconeogenesis	185 (2.13%)	2.75E−01	ko00010	Metabolism
42	Isoquinoline alkaloid biosynthesis	31 (0.36%)	3.55E−01	ko00950	Metabolism
43	ABC transporters	122 (1.41%)	3.71E−01	ko02010	Environmental Information Processing
44	Pentose phosphate pathway	93 (1.07%)	3.78E−01	ko00030	Metabolism
45	Nucleotide excision repair	151 (1.74%)	4.22E−01	ko03420	Genetic Information Processing
46	Riboflavin metabolism	19 (0.22%)	4.22E−01	ko00740	Metabolism
47	Pantothenate and CoA biosynthesis	36 (0.42%)	4.33E−01	ko00770	Metabolism
48	Homologous recombination	138 (1.59%)	4.33E−01	ko03440	Genetic Information Processing
49	Valine, leucine, and isoleucine biosynthesis	31 (0.36%)	4.33E−01	ko00290	Metabolism
50	Phenylalanine, tyrosine, and tryptophan biosynthesis	42 (0.48%)	4.36E−01	ko00400	Metabolism
51	Histidine metabolism	27 (0.31%)	4.36E−01	ko00340	Metabolism
52	Alanine, aspartate, and glutamate metabolism	61 (0.7%)	4.56E−01	ko00250	Metabolism
53	Folate biosynthesis	18 (0.21%)	4.75E−01	ko00790	Metabolism
54	Glycerolipid metabolism	103 (1.19%)	4.86E−01	ko00561	Metabolism
55	Galactose metabolism	96 (1.11%)	4.88E−01	ko00052	Metabolism
56	Carbon fixation in photosynthetic organisms	93 (1.07%)	5.74E−01	ko00710	Metabolism
57	DNA replication	135 (1.56%)	5.80E−01	ko03030	Genetic Information Processing
58	Glycine, serine, and threonine metabolism	67 (0.77%)	6.39E−01	ko00260	Metabolism
59	Insulin resistance	76 (0.88%)	6.90E−01	ko04931	Human Diseases
60	Starch and sucrose metabolism	248 (2.86%)	6.91E−01	ko00500	Metabolism
61	Arginine biosynthesis	46 (0.53%)	6.91E−01	ko00220	Metabolism
62	Sphingolipid metabolism	66 (0.76%)	6.95E−01	ko00600	Metabolism
63	Biosynthesis of unsaturated fatty acids	38 (0.44%)	6.95E−01	ko01040	Metabolism
64	Biosynthesis of amino acids	277 (3.2%)	8.15E−01	ko01230	Metabolism
65	Proteasome	45 (0.52%)	8.16E−01	ko03050	Genetic Information Processing
66	*N*‐Glycan biosynthesis	50 (0.58%)	8.32E−01	ko00510	Metabolism
67	Terpenoid backbone biosynthesis	57 (0.66%)	8.94E−01	ko00900	Metabolism
68	Other types of O‐glycan biosynthesis	15 (0.17%)	9.08E−01	ko00514	Metabolism
69	Fructose and mannose metabolism	70 (0.81%)	9.12E−01	ko00051	Metabolism
70	RNA transport	299 (3.45%)	9.24E−01	ko03013	Genetic Information Processing
71	SNARE interactions in vesicular transport	26 (0.3%)	9.52E−01	ko04130	Genetic Information Processing
72	Phenylpropanoid biosynthesis	125 (1.44%)	9.88E−01	ko00940	Metabolism
73	Other glycan degradation	77 (0.89%)	9.88E−01	ko00511	Metabolism
74	Lysine degradation	46 (0.53%)	9.88E−01	ko00310	Metabolism
75	Citrate cycle (TCA cycle)	48 (0.55%)	9.88E−01	ko00020	Metabolism
76	Synthesis and degradation of ketone bodies	4 (0.05%)	9.88E−01	ko00072	Metabolism
77	Zeatin biosynthesis	32 (0.37%)	9.88E−01	ko00908	Metabolism
78	Cysteine and methionine metabolism	94 (1.08%)	9.88E−01	ko00270	Metabolism
79	Nicotinate and nicotinamide metabolism	26 (0.3%)	9.88E−01	ko00760	Metabolism
80	Glycerophospholipid metabolism	110 (1.27%)	9.88E−01	ko00564	Metabolism
81	Base excision repair	52 (0.6%)	9.88E−01	ko03410	Genetic Information Processing
82	Carbon metabolism	285 (3.29%)	9.88E−01	ko01200	Metabolism
83	Pyruvate metabolism	107 (1.23%)	9.88E−01	ko00620	Metabolism
84	Basal transcription factors	54 (0.62%)	9.88E−01	ko03022	Genetic Information Processing
85	Tropane, piperidine, and pyridine alkaloid biosynthesis	27 (0.31%)	1.00E+00	ko00960	Metabolism
86	Propanoate metabolism	40 (0.46%)	1.00E+00	ko00640	Metabolism
87	2‐Oxocarboxylic acid metabolism	70 (0.81%)	1.00E+00	ko01210	Metabolism
88	Fatty acid elongation	21 (0.24%)	1.00E+00	ko00062	Metabolism
89	beta‐Alanine metabolism	49 (0.57%)	1.00E+00	ko00410	Metabolism
90	Benzoxazinoid biosynthesis	6 (0.07%)	1.00E+00	ko00402	Metabolism
91	Lipoic acid metabolism	3 (0.03%)	1.00E+00	ko00785	Metabolism
92	mRNA surveillance pathway	177 (2.04%)	1.00E+00	ko03015	Genetic Information Processing
93	Sulfur metabolism	36 (0.42%)	1.00E+00	ko00920	Metabolism
94	Phosphatidylinositol signaling system	73 (0.84%)	1.00E+00	ko04070	Environmental Information Processing
95	Monoterpenoid biosynthesis	19 (0.22%)	1.00E+00	ko00902	Metabolism
96	One carbon pool by folate	11 (0.13%)	1.00E+00	ko00670	Metabolism
97	Aminoacyl‐tRNA biosynthesis	73 (0.84%)	1.00E+00	ko00970	Genetic Information Processing
98	Selenocompound metabolism	17 (0.2%)	1.00E+00	ko00450	Metabolism
99	RNA polymerase	51 (0.59%)	1.00E+00	ko03020	Genetic Information Processing
100	Glycosaminoglycan degradation	25 (0.29%)	1.00E+00	ko00531	Metabolism
101	Taurine and hypotaurine metabolism	2 (0.02%)	1.00E+00	ko00430	Metabolism
102	RNA degradation	172 (1.98%)	1.00E+00	ko03018	Genetic Information Processing
103	Regulation of autophagy	49 (0.57%)	1.00E+00	ko04140	Cellular Processes
104	Biotin metabolism	9 (0.1%)	1.00E+00	ko00780	Metabolism
105	Glyoxylate and dicarboxylate metabolism	68 (0.78%)	1.00E+00	ko00630	Metabolism
106	Oxidative phosphorylation	85 (0.98%)	1.00E+00	ko00190	Metabolism
107	Inositol phosphate metabolism	53 (0.61%)	1.00E+00	ko00562	Metabolism
108	Glycosphingolipid biosynthesis—ganglio series	16 (0.18%)	1.00E+00	ko00604	Metabolism
109	Protein export	25 (0.29%)	1.00E+00	ko03060	Genetic Information Processing
110	Glucosinolate biosynthesis	14 (0.16%)	1.00E+00	ko00966	Metabolism
111	Caffeine metabolism	1 (0.01%)	1.00E+00	ko00232	Metabolism
112	Ribosome	161 (1.86%)	1.00E+00	ko03010	Genetic Information Processing
113	Ribosome biogenesis in eukaryotes	129 (1.49%)	1.00E+00	ko03008	Genetic Information Processing
114	Photosynthesis—antenna proteins	11 (0.13%)	1.00E+00	ko00196	Metabolism
115	Sulfur relay system	9 (0.1%)	1.00E+00	ko04122	Genetic Information Processing
116	Glycosphingolipid biosynthesis—globo series	6 (0.07%)	1.00E+00	ko00603	Metabolism
117	Purine metabolism	170 (1.96%)	1.00E+00	ko00230	Metabolism
118	Peroxisome	92 (1.06%)	1.00E+00	ko04146	Cellular Processes
119	Linoleic acid metabolism	15 (0.17%)	1.00E+00	ko00591	Metabolism
120	Photosynthesis	24 (0.28%)	1.00E+00	ko00195	Metabolism
121	Diterpenoid biosynthesis	28 (0.32%)	1.00E+00	ko00904	Metabolism
122	Pyrimidine metabolism	120 (1.38%)	1.00E+00	ko00240	Metabolism
123	Ubiquitin mediated proteolysis	124 (1.43%)	1.00E+00	ko04120	Genetic Information Processing
124	Phagosome	55 (0.63%)	1.00E+00	ko04145	Cellular Processes
125	Glycosylphosphatidylinositol(GPI)‐anchor biosynthesis	9 (0.1%)	1.00E+00	ko00563	Metabolism
126	Thiamine metabolism	3 (0.03%)	1.00E+00	ko00730	Metabolism
127	Endocytosis	279 (3.22%)	1.00E+00	ko04144	Cellular Processes
128	Plant‐pathogen interaction	330 (3.81%)	1.00E+00	ko04626	Organismal Systems
129	Spliceosome	299 (3.45%)	1.00E+00	ko03040	Genetic Information Processing
130	Protein processing in endoplasmic reticulum	261 (3.01%)	1.00E+00	ko04141	Genetic Information Processing
131	Cyanoamino acid metabolism	50 (0.58%)	1.00E+00	ko00460	Metabolism
132	Cutin, suberine and wax biosynthesis	25 (0.29%)	1.00E+00	ko00073	Metabolism
133	Plant hormone signal transduction	223 (2.57%)	1.00E+00	ko04075	Environmental Information Processing

**Table A4:** Summary of high‐throughput sequencing results of *S. canadensis* small RNAs

Types	D1	D2	D3	H1	H2	H3
Total reads	29,794,250	29,769,382	30,089,531	29,659,432	30,538,664	30,082,793
High quality	28,662,008	28,616,221	29,119,621	28,456,348	29,785,812	29,129,419
3′ adapter null	857,422	939,693	774,556	955,551	942,121	1,149,111
Insert null	4,884	4,435	3,161	3,296	11,658	23,691
5′ adapter contaminants	20,936	18,487	25,611	25,329	26,543	30,913
Length small than 18 nt	82,118	249,833	384,772	97,260	332,130	167,214
Poly A	900	3,616	1,191	3,217	2,463	2,386
Clean reads	27,695,748	27,400,157	27,930,330	27,371,695	28,470,897	27,756,104

**Table A5:** List of oppositely regulated miRNA‐target pairs in the transcriptome and sRNA sequencing

MiRNA family	MiRNA	MiRNA log_2_ FC	Target location	Target log_2_ FC	Target function
miR156	sca‐miR156a	−1.12	CL14854.Contig4_All	3.86	Unknown
miR160	sca‐miR160e	4.90	CL5636.Contig1_All	−2.93	Uncharacterized protein LOC104216279 isoform X3
miR161	sca‐miR161a	−1.39	CL11073.Contig3_All	2.62	ATP sulfurylase 2
			Unigene3240_All	7.45	Receptor‐like protein kinase FERONIA
miR164	sca‐miR164d	4.07	Unigene3688_All	−2.14	FAR1
			Unigene13166_All	−4.50	Wall‐associated receptor kinase‐like 6
miR165	sca‐miR165a	1.16	CL3766.Contig4_All	−2.83	Homeobox‐leucine zipper protein REVOLUTA
			CL3766.Contig1_All	−3.40	Homeobox‐leucine zipper protein REVOLUTA
			CL3766.Contig6_All	−8.43	Homeobox‐leucine zipper protein REVOLUTA
miR166	sca‐miR166p	5.16	Unigene5410_All	−7.61	Hypothetical protein AMTR_s00109p00105850
			CL4387.Contig1_All	−3.45	Unknown
			CL2373.Contig3_All	−3.51	Ribosomal protein L5
miR169	sca‐miR169d	−1.24	CL6884.Contig3_All	6.65	Dirigent protein 21
			CL6884.Contig1_All	6.50	Dirigent protein 21
			CL12537.Contig3_All	6.35	Nuclear transcription factor Y subunit A−1
			CL4633.Contig1_All	2.43	Nuclear transcription factor Y subunit A−8
			CL12537.Contig2_All	5.95	Nuclear transcription factor Y subunit A−9
	sca‐miR169e	4.35	CL16645.Contig3_All	−4.51	Calcium‐dependent protein kinase 4
			CL2545.Contig2_All	−2.57	Probable UDP−3‐O‐acylglucosamine *N*‐acyltransferase 2
miR171	sca‐miR171c	2.79	Unigene25276_All	−5.07	Ras‐related protein Rab7
			CL11323.Contig2_All	−4.39	U‐box superfamily protein
miR393	sca‐miR393d	2.48	Unigene1223_All	−2.53	F‐box protein
			CL3203.Contig2_All	−2.48	Transport inhibitor response 1‐like protein
	sca‐miR393e	1.72	CL3203.Contig2_All	−2.48	Transport inhibitor response 1‐like protein
miR396	sca‐miR396a	5.04	CL2872.Contig4_All	−2.13	DEAD‐box ATP‐dependent RNA helicase 42
			CL3408.Contig3_All	−2.20	Glutamate synthase 1
			CL3408.Contig1_All	−6.58	Glutamate synthase 1
			CL6266.Contig7_All	−2.93	Trihelix transcription factor GT−1
	sca‐miR396d	3.50	CL7649.Contig3_All	−3.28	DNA (cytosine−5)‐methyltransferase 1A
			CL7649.Contig4_All	−5.17	DNA (cytosine−5)‐methyltransferase 1A
			Unigene30116_All	−5.53	DNA (cytosine−5)‐methyltransferase 1A
			Unigene21625_All	−6.75	DNA (cytosine−5)‐methyltransferase 1A
			CL7984.Contig4_All	−4.01	Structural maintenance of chromosomes protein 5
miR444	sca‐miR444a	4.18	CL5553.Contig1_All	−2.05	ABC transporter B family member 27
			CL2235.Contig13_All	−7.14	Probable E3 ubiquitin ligase SUD1
			CL2235.Contig14_All	−7.31	Probable E3 ubiquitin ligase SUD1
			CL6747.Contig1_All	−7.57	Transcription factor IIIB 90 kDa subunit
			CL5418.Contig5_All	−5.63	Uncharacterized protein ycf45
			CL5418.Contig11_All	−7.72	Uncharacterized protein ycf45
	sca‐miR444b	3.88	CL1135.Contig2_All	−4.92	Cysteine proteinase RD21a
			CL1135.Contig6_All	−6.70	Hypothetical protein EUGRSUZ_H026191, partial
			CL1135.Contig5_All	−6.98	Unnamed protein product
miR5048	sca‐miR5048a	3.48	Unigene14872_All	−2.57	Auxin‐binding protein T85
miR5139	sca‐miR5139a	−2.10	Unigene17742_All	2.48	Gamma‐glutamyl hydrolase 2
			Unigene7716_All	6.64	HA383 clone BAC 0148N20, complete sequence
			Unigene15641_All	6.64	LRR receptor‐like serine/threonine‐protein kinase
			Unigene5583_All	3.96	Unknown
			CL2157.Contig2_All	4.05	Oryza sativa genomic DNA
			CL5222.Contig1_All	2.77	Retrovirus‐related Pol polyprotein from transposon TNT 1–94
			CL17163.Contig1_All	2.05	Retrovirus‐related Pol polyprotein from transposon TNT 1–94
			CL1303.Contig1_All	5.73	Transcription factor VOZ1‐like
			CL2207.Contig4_All	6.41	U‐box domain‐containing protein 30
	sca‐miR5139b	−1.87	Unigene26373_All	3.19	Anthocyanidin 5,3‐O‐glucosyltransferase
			Unigene7716_All	6.64	HA383 clone BAC 0148N20, complete sequence
			Unigene15641_All	6.64	LRR receptor‐like serine/threonine‐protein kinase
			CL2157.Contig2_All	4.05	Oryza sativa genomic DNA
			CL17163.Contig1_All	2.05	Retrovirus‐related Pol polyprotein from transposon TNT 1–94
			CL2207.Contig4_All	6.41	U‐box domain‐containing protein 30
miR530	sca‐miR530	−1.13	Unigene14091_All	5.08	Probable disease resistance protein
			CL1613.Contig1_All	5.74	Transcription factor MYB1R1
miR6173	sca‐miR6173	−2.45	CL13158.Contig1_All	3.25	Protein YLS9
miR6300	sca‐miR6300	−1.02	Unigene8614_All	2.59	Flowering time control protein FCA
			CL3059.Contig5_All	5.32	Gag‐pol polyprotein
			Unigene7996_All	4.80	Laccase−15
miR8155	sca‐miR8155	−3.15	Unigene4360_All	3.97	ABC transporter D family member 1
			Unigene16624_All	5.45	ABC transporter G family member 31
			CL4044.Contig2_All	3.13	Cysteine synthase
			Unigene2778_All	6.69	Delta−1‐pyrroline−5‐carboxylate dehydrogenase 1 protein
			Unigene6077_All	4.95	Delta−1‐pyrroline−5‐carboxylate dehydrogenase 1 protein
			Unigene17742_All	2.48	Gamma‐glutamyl hydrolase 2
			Unigene7716_All	6.64	HA383 clone BAC 0148N20, complete sequence
			Unigene15641_All	6.64	LRR receptor‐like serine/threonine‐protein kinase
			Unigene5583_All	3.96	Unknown
			CL2157.Contig2_All	4.05	Oryza sativa genomic DNA
			CL10305.Contig2_All	9.88	Probable pyridoxal biosynthesis protein PDX1.2
			CL3399.Contig2_All	7.36	Protein ECERIFERUM 3
			CL5222.Contig1_All	2.77	Retrovirus‐related Pol polyprotein from transposon TNT 1–94
			CL17163.Contig1_All	2.05	Retrovirus‐related Pol polyprotein from transposon TNT 1–94
			CL2207.Contig4_All	6.41	U‐box domain‐containing protein 30
miR894	sca‐miR894	−2.09	CL732.Contig5_All	6.38	Hypothetical protein MTR_4g131890
			CL732.Contig8_All	6.12	Hypothetical protein MTR_4g131890
			CL732.Contig4_All	7.09	Unknown
miR9662	sca‐miR9662a	3.45	CL14321.Contig1_All	−3.13	Mitochondrial import inner membrane translocase subunit TIM10

**Table A6:** List of differentially expressed unigenes associated with CYPs

Unigene	ID	Plausible metabolic pathway	D_FPKM	H_FPKM	Log**_2 _**FC	Up/down
CL7441.Contig4_All	CYP59		0.85	52.82	5.95	Up
Unigene11828_All	CYP19‐4		20.74	380.99	4.20	Up
CL13176.Contig1_All	CYP19‐4		16.40	165.24	3.33	Up
CL10739.Contig3_All	CYP19‐4		67.91	477.41	2.81	Up
CL13176.Contig2_All	CYP19‐4		133.98	27.73	−2.27	Down
Unigene6217_All	CYP19‐4		174.06	22.41	−2.96	Down
Unigene30406_All	CYP19‐4		33.84	3.09	−3.45	Down
CL10739.Contig1_All	CYP19‐4		216.00	1.45	−7.22	Down
CL9185.Contig2_All	CYP2		85.28	4,912.78	5.85	Up
CL9185.Contig1_All	CYP2		6,998.33	984.99	−2.83	Down
CL10739.Contig2_All	CYP20‐3		30.98	4.35	−2.83	Down
CL9682.Contig2_All	CYP21‐3		122.88	28.86	−2.09	Down
CL2380.Contig5_All	CYP40		15.43	261.52	4.08	Up
Unigene2002_All	CYP40		16.43	71.43	2.12	Up
CL7441.Contig2_All	CYP59		89.44	17.15	−2.38	Down
Unigene29249_All	CYP63		24.86	0.67	−5.22	Down
Unigene22411_All	CYP704C1	Stilbenoid, diarylheptanoid, and gingerol biosynthesis	86.83	1,208.25	3.80	Up
Unigene22213_All	CYP704C1	Stilbenoid, diarylheptanoid, and gingerol biosynthesis	17.65	214.89	3.61	Up
CL1310.Contig1_All	CYP704C1	Cutin, suberine, and wax biosynthesis	78.69	914.18	3.54	Up
Unigene22236_All	CYP704C1	Stilbenoid, diarylheptanoid, and gingerol biosynthesis	144.79	19.62	−2.88	Down
Unigene5770_All	CYP704C1	Stilbenoid, diarylheptanoid, and gingerol biosynthesis	121.73	5.83	−4.38	Down
CL14169.Contig2_All	CYP710A1		68.67	319.20	2.22	Up
CL4539.Contig3_All	CYP711A1		51.23	4.49	−3.51	Down
CL2023.Contig1_All	CYP716B1		631.08	32.09	−4.30	Down
CL2023.Contig3_All	CYP716B1		1,621.08	31.09	−5.70	Down
CL2023.Contig7_All	CYP716B2		15.01	1,730.37	6.85	Up
Unigene12692_All	CYP71A1	Flavone and flavonol biosynthesis;Flavonoid biosynthesis	90.37	8.93	−3.34	Down
Unigene15825_All	CYP71A2	Stilbenoid, diarylheptanoid, and gingerol biosynthesis	2.13	140.71	6.05	Up
CL8060.Contig1_All	CYP71A2	Stilbenoid, diarylheptanoid, and gingerol biosynthesis	898.29	8,213.10	3.19	Up
CL977.Contig3_All	CYP71A4	Stilbenoid, diarylheptanoid, and gingerol biosynthesis	183.01	1,254.69	2.78	Up
CL977.Contig2_All	CYP71A4	Stilbenoid, diarylheptanoid, and gingerol biosynthesis	32.82	197.65	2.59	Up
CL977.Contig5_All	CYP71A4	Stilbenoid, diarylheptanoid, and gingerol biosynthesis	143.64	776.66	2.43	Up
Unigene26266_All	CYP71A4	Stilbenoid, diarylheptanoid, and gingerol biosynthesis	80.86	2.90	−4.80	Down
Unigene17018_All	CYP71A4		275.49	4.65	−5.89	Down
CL15273.Contig1_All	CYP71A6	Stilbenoid, diarylheptanoid, and gingerol biosynthesis	5.13	620.22	6.92	Up
CL15955.Contig1_All	CYP71A8	Stilbenoid, diarylheptanoid, and gingerol biosynthesis	2.73	331.81	6.92	Up
CL8060.Contig3_All	CYP71AJ1	Stilbenoid, diarylheptanoid, and gingerol biosynthesis	19.87	827.24	5.38	Up
Unigene23908_All	CYP71AV1		23.88	0.29	−6.36	Down
CL6714.Contig2_All	CYP71AV8	Sesquiterpenoid and triterpenoid biosynthesis	0.27	28.54	6.71	Up
CL6714.Contig1_All	CYP71AV8	Sesquiterpenoid and triterpenoid biosynthesis	82.83	1.05	−6.30	Down
CL9888.Contig1_All	CYP71BL3		26.09	0.29	−6.48	Down
Unigene23159_All	CYP71D55	Sesquiterpenoid and triterpenoid biosynthesis	64.86	8.37	−2.95	Down
CL15279.Contig1_All	CYP71D55	Sesquiterpenoid and triterpenoid biosynthesis	715.14	75.17	−3.25	Down
CL9608.Contig2_All	CYP71D55	Sesquiterpenoid and triterpenoid biosynthesis	423.22	28.36	−3.90	Down
CL5543.Contig2_All	CYP72A154		7.85	200.64	4.67	Up
Unigene27925_All	CYP72A219		0.23	15.61	6.09	Up
Unigene22475_All	CYP72A219		2.61	85.74	5.04	Up
CL4064.Contig5_All	CYP72A219		27.03	389.02	3.85	Up
Unigene6002_All	CYP72A219		39.16	198.43	2.34	Up
CL10015.Contig2_All	CYP72A219		370.63	91.20	−2.02	Down
Unigene6055_All	CYP72A219		1,306.33	209.38	−2.64	Down
Unigene5550_All	CYP72A219		1,484.07	76.13	−4.29	Down
CL553.Contig1_All	CYP72A219		17.50	0.65	−4.76	Down
CL4046.Contig2_All	CYP749A22	Brassinosteroid biosynthesis	0.30	77.92	8.00	Up
CL4046.Contig8_All	CYP749A22	Brassinosteroid biosynthesis	0.25	18.51	6.21	Up
CL4046.Contig1_All	CYP749A22	Brassinosteroid biosynthesis	8.29	138.16	4.06	Up
CL14877.Contig1_All	CYP749A22	Brassinosteroid biosynthesis	38.24	315.95	3.05	Up
CL4046.Contig5_All	CYP749A22	Brassinosteroid biosynthesis	24.46	0.32	−6.26	Down
CL4046.Contig6_All	CYP749A22	Brassinosteroid biosynthesis	203.05	1.08	−7.55	Down
CL37.Contig2_All	CYP75B2	Flavone and flavonol biosynthesis;Flavonoid biosynthesis	1.58	630.70	8.64	Up
CL8453.Contig3_All	CYP75B2		465.26	90.17	−2.37	Down
CL37.Contig1_All	CYP75B2	Flavone and flavonol biosynthesis;Flavonoid biosynthesis	708.39	23.03	−4.94	Down
CL1605.Contig12_All	CYP76AD1		118.74	26.26	−2.18	Down
CL2830.Contig3_All	CYP76B1	Flavonoid biosynthesis;Stilbenoid, diarylheptanoid and gingerol biosynthesis;Phenylpropanoid biosynthesis	3.67	82.10	4.48	Up
CL3689.Contig2_All	CYP76B6	Flavone and flavonol biosynthesis;Flavonoid biosynthesis	3.79	65.45	4.11	Up
CL1852.Contig2_All	CYP76B6	Flavone and flavonol biosynthesis;Flavonoid biosynthesis	42.67	171.06	2.00	Up
CL4701.Contig3_All	CYP76C1		32.81	195.06	2.57	Up
Unigene28192_All	CYP76C1		93.38	421.32	2.17	Up
CL7793.Contig1_All	CYP76C1		136.81	560.21	2.03	Up
CL7793.Contig2_All	CYP76C1		1,323.87	213.51	−2.63	Down
CL4701.Contig5_All	CYP76C1		24.44	1.49	−4.04	Down
CL4701.Contig4_All	CYP76C1		79.49	0.33	−7.89	Down
CL14510.Contig2_All	CYP77A2	Stilbenoid, diarylheptanoid, and gingerol biosynthesis	59.18	900.01	3.93	Up
CL5637.Contig1_All	CYP79D1	Glucosinolate biosynthesis	1.58	43.17	4.77	Up
CL8279.Contig1_All	CYP79D1	Glucosinolate biosynthesis	39.87	6.14	−2.70	Down
CL2748.Contig1_All	CYP80B2		1.46	30.68	4.40	Up
CL9352.Contig4_All	CYP81D1	Stilbenoid, diarylheptanoid, and gingerol biosynthesis	57.61	8.79	−2.71	Down
CL8387.Contig2_All	CYP81E1	Stilbenoid, diarylheptanoid, and gingerol biosynthesis	90.19	15.56	−2.54	Down
CL13045.Contig1_All	CYP81E1	Stilbenoid, diarylheptanoid, and gingerol biosynthesis	39.39	1.06	−5.22	Down
CL13045.Contig2_All	CYP81E1	Stilbenoid, diarylheptanoid, and gingerol biosynthesis	54.59	0.67	−6.36	Down
Unigene7285_All	CYP82A3	Stilbenoid, diarylheptanoid, and gingerol biosynthesis	0.27	48.54	7.47	Up
Unigene5205_All	CYP82A3	Stilbenoid, diarylheptanoid, and gingerol biosynthesis	0.26	43.78	7.40	Up
Unigene4201_All	CYP82A3	Stilbenoid, diarylheptanoid, and gingerol biosynthesis	0.25	24.18	6.58	Up
Unigene4771_All	CYP82A3	Stilbenoid, diarylheptanoid, and gingerol biosynthesis	0.88	39.34	5.47	Up
CL14937.Contig5_All	CYP82A3	Stilbenoid, diarylheptanoid, and gingerol biosynthesis	30.52	375.55	3.62	Up
CL10835.Contig1_All	CYP82A3	Stilbenoid, diarylheptanoid, and gingerol biosynthesis	4.99	55.93	3.49	Up
CL2495.Contig1_All	CYP82A3	Stilbenoid, diarylheptanoid, and gingerol biosynthesis	5.87	63.33	3.43	Up
CL17044.Contig1_All	CYP82A3	Stilbenoid, diarylheptanoid, and gingerol biosynthesis	795.11	168.53	−2.24	Down
Unigene25796_All	CYP82A3	Stilbenoid, diarylheptanoid, and gingerol biosynthesis	500.03	65.44	−2.93	Down
CL8654.Contig1_All	CYP82A3	Stilbenoid, diarylheptanoid, and gingerol biosynthesis	94.77	10.61	−3.16	Down
CL3118.Contig2_All	CYP82G1	Diterpenoid biosynthesis	23.59	3,287.45	7.12	Up
CL3531.Contig2_All	CYP83B1	Glucosinolate biosynthesis	0.70	110.50	7.29	Up
Unigene28320_All	CYP84A1	Phenylpropanoid biosynthesis	453.58	78.83	−2.52	Down
CL2645.Contig3_All	CYP85A1	Brassinosteroid biosynthesis	1.90	31.87	4.07	Up
CL16982.Contig1_All	CYP85A1	Brassinosteroid biosynthesis	68.52	2.80	−4.62	Down
CL16982.Contig2_All	CYP85A1	Brassinosteroid biosynthesis	32.06	0.30	−6.74	Down
Unigene9790_All	CYP86A8	Cutin, suberine, and wax biosynthesis	8.85	121.08	3.77	Up
CL13254.Contig3_All	CYP86B1	Cutin, suberine, and wax biosynthesis	4.35	472.95	6.76	Up
Unigene17254_All	CYP86B1	Cutin, suberine, and wax biosynthesis	32.44	167.27	2.37	Up
CL2327.Contig3_All	CYP87A3		26.15	0.33	−6.31	Down
CL6582.Contig6_All	CYP89A2	Stilbenoid, diarylheptanoid, and gingerol biosynthesis	4.48	267.01	5.90	Up
CL10339.Contig2_All	CYP90A1	Brassinosteroid biosynthesis	7.21	80.63	3.48	Up
CL10339.Contig3_All	CYP90A1	Brassinosteroid biosynthesis	456.50	107.30	−2.09	Down
CL361.Contig6_All	CYP93A1	Isoflavonoid biosynthesis	46.89	4.48	−3.39	Down
CL1330.Contig3_All	CYP93A3	Isoflavonoid biosynthesis	0.57	41.79	6.20	Up
CL16738.Contig3_All	CYP93A3	Isoflavonoid biosynthesis	70.10	924.85	3.72	Up
CL16738.Contig1_All	CYP93A3	Isoflavonoid biosynthesis	46.85	269.17	2.52	Up
CL1330.Contig7_All	CYP93A3	Isoflavonoid biosynthesis	120.62	12.75	−3.24	Down
CL1330.Contig6_All	CYP93A3	Isoflavonoid biosynthesis	148.28	12.08	−3.62	Down
CL7523.Contig2_All	CYP94A1	Stilbenoid, diarylheptanoid, and gingerol biosynthesis	1.22	142.26	6.87	Up
CL2760.Contig1_All	CYP94C1	Cutin, suberine, and wax biosynthesis	2.94	163.00	5.79	Up
CL5348.Contig1_All	CYP94C1		33.97	0.30	−6.82	Down
CL5348.Contig2_All	CYP94C1		76.68	0.34	−7.84	Down
CL4017.Contig2_All	CYP95		3.14	68.85	4.45	Up
Unigene35557_All	CYP97A3	Carotenoid biosynthesis	0.35	469.90	10.40	Up
Unigene9891_All	CYP97B2	Carotenoid biosynthesis	53.06	508.17	3.26	Up
Unigene9890_All	CYP97B2	Carotenoid biosynthesis	219.04	47.55	−2.20	Down
CL3333.Contig5_All	CYP97B2	Carotenoid biosynthesis	78.73	13.35	−2.56	Down
Unigene21607_All	CYP97B2	Carotenoid biosynthesis	428.64	0.41	−10.04	Down
CL1848.Contig1_All	CYP98A2	Flavonoid biosynthesis;Stilbenoid, diarylheptanoid and gingerol biosynthesis;Phenylpropanoid biosynthesis	44.29	3.11	−3.83	Down

**Table A7:** List of differentially expressed unigenes associated with UGTs

Unigene	Unigene description	ID	D_FPKM	H_FPKM	Log_2 _FC	Up/down
CL3889.Contig1_All	Anthocyanidin 3‐O‐glucosyltransferase	RT	121.76	5.30	−4.52	Down
CL3889.Contig2_All	Anthocyanidin 3‐O‐glucosyltransferase	RT	297.22	4.92	−5.92	Down
CL3889.Contig3_All	Anthocyanidin 3‐O‐glucosyltransferase	RT	232.97	2.63	−6.47	Down
CL3889.Contig4_All	Anthocyanidin 3‐O‐glucosyltransferase	RT	321.72	3.03	−6.73	Down
CL373.Contig2_All	Anthocyanidin 5,3‐O‐glucosyltransferase	RhGT1	63.22	381.10	2.59	Up
CL373.Contig1_All	Anthocyanidin 5,3‐O‐glucosyltransferase	RhGT1	59.30	241.43	2.03	Up
Unigene19820_All	Scopoletin glucosyltransferase	TOGT1	174.28	754.95	2.12	Up
CL6939.Contig2_All	Sterol 3‐beta‐glucosyltransferase	UGT80B1	396.44	84.58	−2.23	Down
CL5245.Contig8_All	Sterol 3‐beta‐glucosyltransferase UGT80A2	UGT80A2	0.37	47.22	6.98	Up
Unigene16792_All	Sterol 3‐beta‐glucosyltransferase UGT80A2	UGT80A2	0.27	20.82	6.27	Up
Unigene16787_All	Sterol 3‐beta‐glucosyltransferase UGT80A2	UGT80A2	13.97	75.97	2.44	Up
Unigene16789_All	Sterol 3‐beta‐glucosyltransferase UGT80A2	UGT80A2	35.83	2.35	−3.93	Down
Unigene16790_All	Sterol 3‐beta‐glucosyltransferase UGT80A2	UGT80A2	81.30	0.33	−7.93	Down
CL6939.Contig1_All	Sterol 3‐beta‐glucosyltransferase UGT80B1	UGT80B1	9.07	181.65	4.32	Up
CL6939.Contig5_All	Sterol 3‐beta‐glucosyltransferase UGT80B1	UGT80B1	18.82	138.67	2.88	Up
CL9172.Contig2_All	UDP‐glucose flavonoid 3‐O‐glucosyltransferase 6	GT6	4.29	82.32	4.26	Up
CL13096.Contig1_All	UDP‐glycosyltransferase 73C3	UGT73C3	470.86	56.54	−3.06	Down
Unigene795_All	UDP‐glycosyltransferase 73C3	UGT73C3	33.30	3.70	−3.17	Down
CL5682.Contig4_All	UDP‐glycosyltransferase 73C5	UGT73C5	101.33	1,257.38	3.63	Up
Unigene22107_All	UDP‐glycosyltransferase 73C5	UGT73C5	1,799.31	358.74	−2.33	Down
Unigene748_All	UDP‐glycosyltransferase 73C6	UGT73C6	33.10	190.30	2.52	Up
CL12706.Contig2_All	UDP‐glycosyltransferase 74B1	UGT74B1	1.82	233.31	7.00	Up
CL3968.Contig2_All	UDP‐glycosyltransferase 74E2	UGT74E2	1.50	65.68	5.46	Up
CL3968.Contig1_All	UDP‐glycosyltransferase 74E2	UGT74E2	10.66	158.42	3.89	Up
CL307.Contig2_All	UDP‐glycosyltransferase 76C1	UGT76C1	24.06	1,960.82	6.35	Up
CL307.Contig1_All	UDP‐glycosyltransferase 76C1	UGT76C1	80.06	376.71	2.23	Up
CL4544.Contig1_All	UDP‐glycosyltransferase 76C1	UGT76C1	45.19	9.01	−2.33	Down
Unigene240_All	UDP‐glycosyltransferase 76C2	UGT76C2	1.21	48.70	5.33	Up
CL14018.Contig2_All	UDP‐glycosyltransferase 76C3	UGT76C3	2.47	30.65	3.64	Up
Unigene24228_All	UDP‐glycosyltransferase 76E4	UGT76E4	0.33	99.32	8.21	Up
Unigene52237_All	UDP‐glycosyltransferase 76E4	UGT76E4	0.70	125.56	7.49	Up
CL2638.Contig3_All	UDP‐glycosyltransferase 76E4	UGT76E4	0.34	32.57	6.57	Up
Unigene37400_All	UDP‐glycosyltransferase 76E4	UGT76E4	1.07	44.55	5.39	Up
CL10124.Contig2_All	UDP‐glycosyltransferase 83A1	UGT83A1	49.76	303.61	2.61	Up
CL10124.Contig1_All	UDP‐glycosyltransferase 83A1	UGT83A1	35.29	211.30	2.58	Up
CL11684.Contig1_All	UDP‐glycosyltransferase 83A1	UGT83A1	520.60	99.07	−2.39	Down
CL4313.Contig2_All	UDP‐glycosyltransferase 83A1	UGT83A1	384.98	52.03	−2.89	Down
CL11650.Contig2_All	UDP‐glycosyltransferase 83A1	UGT83A1	70.32	6.45	−3.45	Down
CL3790.Contig2_All	UDP‐glycosyltransferase 85A1	UGT85A1	33.94	139.57	2.04	Up
CL7944.Contig1_All	UDP‐glycosyltransferase 85A2	UGT85A2	0.32	96.90	8.26	Up
CL12789.Contig1_All	UDP‐glycosyltransferase 85A2	UGT85A2	6.27	81.53	3.70	Up
CL9178.Contig2_All	UDP‐glycosyltransferase 85A2	UGT85A2	16.84	145.43	3.11	Up
CL12789.Contig2_All	UDP‐glycosyltransferase 85A2	UGT85A2	33.46	0.68	−5.62	Down
CL10565.Contig2_All	UDP‐glycosyltransferase 85A3	UGT85A3	75.20	312.18	2.05	Up
CL9310.Contig2_All	UDP‐glycosyltransferase 85A5	UGT85A5	49.82	11.55	−2.11	Down
CL3105.Contig3_All	UDP‐glycosyltransferase 85A5	UGT85A5	56.96	10.49	−2.44	Down
Unigene14793_All	UDP‐glycosyltransferase 87A2	UGT87A2	0.61	158.97	8.02	Up
Unigene10460_All	UDP‐glycosyltransferase 89B1	UGT89B1	3.80	102.43	4.75	Up
Unigene4493_All	UDP‐glycosyltransferase 91A1	UGT91A1	30.28	245.03	3.02	Up
CL14041.Contig1_All	UDP‐glycosyltransferase 92A1	UGT92A1	190.22	31.04	−2.62	Down
CL8978.Contig2_All	Zeatin O‐xylosyltransferase	ZOX1	358.94	77.03	−2.22	Down
