# Supplementary material for: Molecular basis underlying the successful invasion of hexaploid cytotypes of Solidago canadensis L.: Insights from integrated gene and miRNA expression profiling
Source: Ecol Evol. 2019 Mar 26;9(8):4820–52. doi: 10.1002/ece3.5084 (PMC6476842; doi:10.1002/ece3.5084)
Supplement: Supplementary file 1 [file ECE3-9-4820-s001.docx]

**Appendix S1** Additional methods

**Chromosome counting**

Chromosome counting was examined according to the modified carbol fuchsin squash method. That is, excised root tips were pretreated in saturated p-dichlorobenezene, and fixed in a freshly-prepared Carnoy’s solution. Subsequently, the fixed root tips were disintegrated with 1% HCl solution at 60°C, and then softened root tips were stained with modified carbol fuchsin, squeezed and microscopic examined. Chromosome photographs with well dispersed metaphases were taken on a Olympus BX-51 microscope by employing DP2BSW software.

**cDNA and small RNA library construction and** **Illumina sequencing**

cDNA library was constructed following the methods provided by Beijing Genomics Institute (BGI, Shenzhen, China). In brief, total RNA was extracted with Trizol reagent (Invitrogen, USA) and digested with RNase-free DNase I. Poly (A)-containing mRNA was enriched from total RNA by using oligo (dT)-conjugated magnetic beads. Then, a fragmentation buffer was used to cleave mRNAs into short fragments, which were used as templates for first-strand cDNA synthesis with random hexamer primers. Second-strand cDNA was synthesized by building second-strand synthesis reaction system including SuperScript II, buffer, dNTPs, RNaseH and DNA polymerase I. Next, short double cDNA fragments were purified with the kit and resolved with EB buffer for cohesive end reparation followed by poly (A) addition and Illumina’s paired end adaptors ligation. After that, suitable fragments were selected as templates for PCR amplification based on an agarose gel electrophoresis and to construct final cDNA libraries. Finally, six qualified cDNA libraries were subjected to deep sequencing on an Illumina HiSeq 4000 platform with 150 bp paired-end reads at BGI.

Total RNA from the same leaf tissues were also used for six small RNA library constructions and sequencing. For each library, 1 µg of total RNA was used to construct a small RNA library for sequencing. In brief, sRNA molecules between 18 and 30 nt were isolated, purified from a 15% denaturing polyacrylamide gel electrophoresis (PAGE) gel, and ligated with 5'- and 3'-adaptors by T4 RNA ligase. Reverse transcription followed by PCR was performed to create cDNA products with adaptor-specific RT-primers. Subsequently, amplification products were purified from a PAGE gel for deep sequencing on a BGISEQ-500 sequencer (BGI, Shenzhen, China).

**Unigene annotation**

The ‘All-Unigene’ sequences were aligned by BLASTx to a series of protein databases such as the National Center for Biotechnology Information nonredundant (Nr), Kyoto Encyclopedia of Genes and Genomes (KEGG), Clusters of Orthologous Groups (COG) and SwissProt protein database and aligned by BLASTn to the nonredundant nucleotide database (Nt) to gain unigene annotation. Based on Nr annotation, gene ontology (GO) functional classifications associated with cellular components, molecular functions and biological processes for the unigenes were obtained via the Blast2-GO program (version: v2.5.0), and InterProScan5 (version: v5.11-51.0) was used to obtain InterPro annotations.

**qRT-PCR**

qRT-PCR reactions were implemented in triplicate on an ABI Step One Plus Real-Time PCR System (Applied Biosystems) in each 20 µl volume including 1 µl diluent complementary DNA, 0.4 µl ROX reference dye, 10 µl Thunderbird SYBR® Green PCR Master mix (Toyobo, Kita-ku, Osaka, Japan), and 0.1 µM of each unigene- and miRNA-specific sense and anti-sense primer (Supporting Information Table S1) with the following two-step cycling programs: 95^o^C hold for 10 min, 40 cycles at 95^o^C for 15 s and 60^o^C for 1 min. With that, a thermal denaturing cycle of 95^o^C for 15 s, 60^o^C for 1 min, and 95^o^C for 15 s was performed to generate melting curves and authenticate the specificity and identity of PCR amplification products.
